# Small things matter: Lack of extraislet β cells in type 1 diabetes

**DOI:** 10.1126/sciadv.adz2251

**Published:** 2025-11-12

**Authors:** Kathryn Murrall, Teifion Luckett, Christiana Lekka, Christine S. Flaxman, Rebecca Wyatt, Pouria Akhbari, Irina Kusmartseva, Stephanie L. Hunter, Pia Leete, Isabel Burn, Elena Osokina, EXE-T1D Consortium, James A. M. Shaw, Noel G. Morgan, Sarah J. Richardson

**Affiliations:** ^1^ Islet Biology Exeter (IBEx), Exeter Centre of Excellence for Diabetes Research (EXCEED), Department of Clinical and Biomedical Sciences, University of Exeter Medical School, Exeter, UK.; ^2^ Indica Labs, Albuquerque, NM 87114, USA.; ^3^ Department of Pathology, Immunology, and Laboratory Medicine, Diabetes Institute, College of Medicine, University of Florida, Gainesville, FL 32611, USA.; ^4^ Cambridge Institute for Therapeutic Immunology and Infectious Disease (CITIID), University of Cambridge, Cambridge, UK.; ^5^ Translational and Clinical Research Institute, Newcastle University, Newcastle upon Tyne, UK.

## Abstract

Recent three-dimensional (3D) analyses reported an abundance of small β cell–rich endocrine objects (EOs) in the human pancreas. Here, we used archival, immunolabeled 2D pancreas sections to assess morphological EO parameters in donors with or without type 1 diabetes (T1D), varying in age and disease duration. We confirm that abundant small, insulin-positive EOs are present in donors without diabetes and comprise most of the pancreatic endocrine area in early life. Small EOs are virtually absent in individuals with T1D, and this effect is most pronounced in children diagnosed with T1D in their earliest years. We conclude that extraislet β cells are affected in T1D development, and their early loss is a characteristic feature. This finding has important implications, which will inform future screening and treatment strategies for T1D.

## Teaser

Early loss of small extraislet β cells in children with type 1 diabetes could explain age-related differences in disease.

## INTRODUCTION

Type 1 diabetes (T1D) is an autoimmune disease in which auto-reactive immune cells selectively target pancreatic β cells, leading to insulin (Ins) insufficiency. The incidence of T1D is rising globally, with 16.4 million people predicted to be affected by 2040 (t1index.org). Increasingly recognized as a condition with equivalent rates of onset in children and adults (*1*, *2*), T1D affects individuals living with the condition, their families, and global healthcare systems. Hence, there is a pressing need to improve the treatment and management of the condition. Previous studies identified heterogeneity in the aetiopathology of T1D linked to age at onset, and in the clinical course of the disease, both of which are likely to affect therapeutic strategies (*3*–*6*).

Global initiatives aimed at improving the understanding of underlying disease mechanisms in T1D recognize the need for systematic collection and collation of pancreas tissue from individuals with, or at risk of developing, T1D and individuals without diabetes (ND) (*7*, *8*). The understanding that human pancreas development (*9*) and T1D (*10*) are not fully recapitulated in rodent models emphasizes the importance of this endeavor. Several biobanks are established, which facilitate the application of multiomic two-dimensional (2D) and 3D histological approaches to explore pancreas growth and development during the life course; however, most focus on fetal or adolescent and adult tissues (*11*–*14*). The recent establishment of the Human Neonatal Development & Early Life Pancreas Atlas seeks to address this, but there remains a substantial gap in our knowledge regarding pancreas development prepuberty, particularly in the first 2 years of life.

A recent study exploited advances in 3D imaging modalities to explore volumetric endocrine mass throughout the entire pancreas of adult human ND donors (*15*), revealing two particularly notable findings. First, 40 to 50% of the islets are small (defined by the authors as <115-μm diameter) and do not conform to the classical islet structure (*15*). Second, and in confirmation of earlier work from a small number of donors (*16*–*20*), most small islets (defined as smaller than 60- to 115-μm diameter by various authors) are composed of β cells (*16*–*20*). However, most histological studies have focused on medium and large structures, leaving these small islets underexplored. Furthermore, functional, transcriptional, and proteomic studies performed using isolated human islets do not provide any insights into the single cells and small clusters, as these are lost during the isolation process (*21*). Consequently, there is limited information about whether islet size and composition change during normal early-life development and with T1D. We now address these knowledge gaps by characterizing the endocrine object (EO; defined as contiguous structures containing one or more endocrine cells) profile in the human pancreas throughout early development, and in T1D, using immunohistochemically labeled tissue sections from archival and contemporary pancreas biobanks.

## RESULTS

### Artificial intelligence–assisted image analysis allows accurate quantification of changes in pancreas architecture and composition

**Fig. 1.**
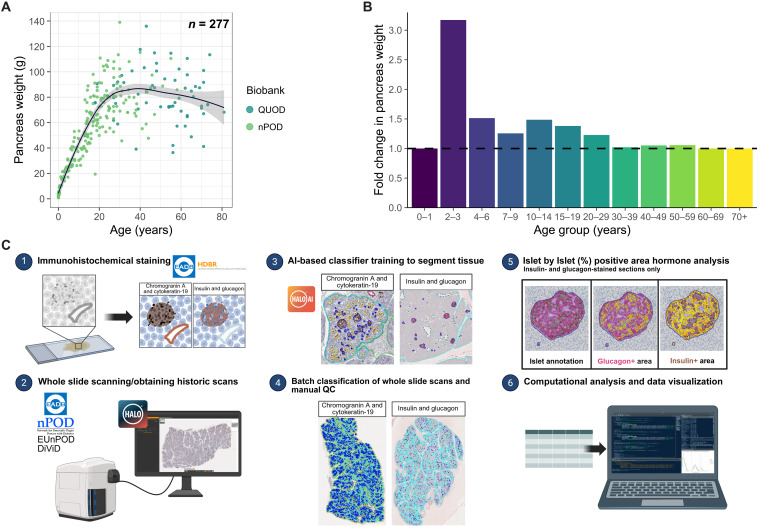
AI-assisted WSI analysis performed to investigate the development of the endocrine architecture postbirth during pancreas expansion. (**A**) Scatter plot of pancreas weight with age postbirth with locally estimated scatterplot smoothing regression line in nPOD and QUOD donors (*n* = 277). (**B**) Fold change in median pancreas weight relative to the previous age bracket. (**C**) WSI analysis methodology and data processing of slides labeled with either CgA and CK19 or Ins and Gluc.

Two organ donor pancreas biobanks exist where pancreas weight is available from donors collected throughout the life course: Network for Pancreatic Organ Donors with Diabetes (nPOD) and Quality in Organ Donation (QUOD-PANC). Combining these datasets confirms that the pancreas grows continuously over the first 30 years of life, after which pancreas weight is relatively stable (Fig. 1A). When comparing fold change in pancreas weight with increasing age, the most pronounced expansion occurs within the first 2 to 3 years (Fig. 1B).

To explore pancreatic architectural changes at greater resolution, we accessed routinely labeled pancreas tissue slides from multiple archival and contemporary bioresources, covering the entire life course [Human Developmental Biology Resource (HDBR), Exeter Archival Diabetes Biobank (EADB), nPOD, Diabetes Virus Detection (DiViD), and European nPOD (EUnPOD)]. We established artificial intelligence (AI)–assisted image analysis pipelines (Fig. 1C) to interrogate the immunolabeling of chromogranin A (CgA; to identify endocrine cells) and cytokeratin-19 (CK19) or combinations of insulin (Ins; to identify β cells) and glucagon (Gluc; to identify α cells). Counterstaining of the sections with hematoxylin allowed visualization of other pancreatic compartments (e.g., acinar, duct, and blood vessels). In total, we analyzed 458 whole slide images (WSI) across 250 donors (CgA and CK19 dataset, *n* = 45; Ins and Gluc dataset, *n* = 205). Individual donor demographics are included in table S1, and associated pancreas section information can be found in table S2. We identified 407,794 EOs in both recently labeled and archival sections. The established pipelines and outputs are described in Fig. 1C and Materials and Methods. This approach yielded data derived from archival donors from whom no additional pancreas tissue is available, as well as from pancreata held in contemporary collections.

Key pipeline outputs included pancreas section area, total endocrine area (and proportion of total tissue area), EO area (in square micrometers) and count, β and α cell area and proportion, and acinar area and proportion. This quality-controlled pipeline allowed the assessment of pancreas architecture throughout the life course (0 to 72 years) in individuals with differing disease status [no diabetes, autoantibody-positive (AAb^+^) without diabetes, and type 1 diabetes (T1D)].

### EO density and area decrease during the first 10 years of life in healthy donors, and the EO profile shifts toward larger islets with age

**Fig. 2.**
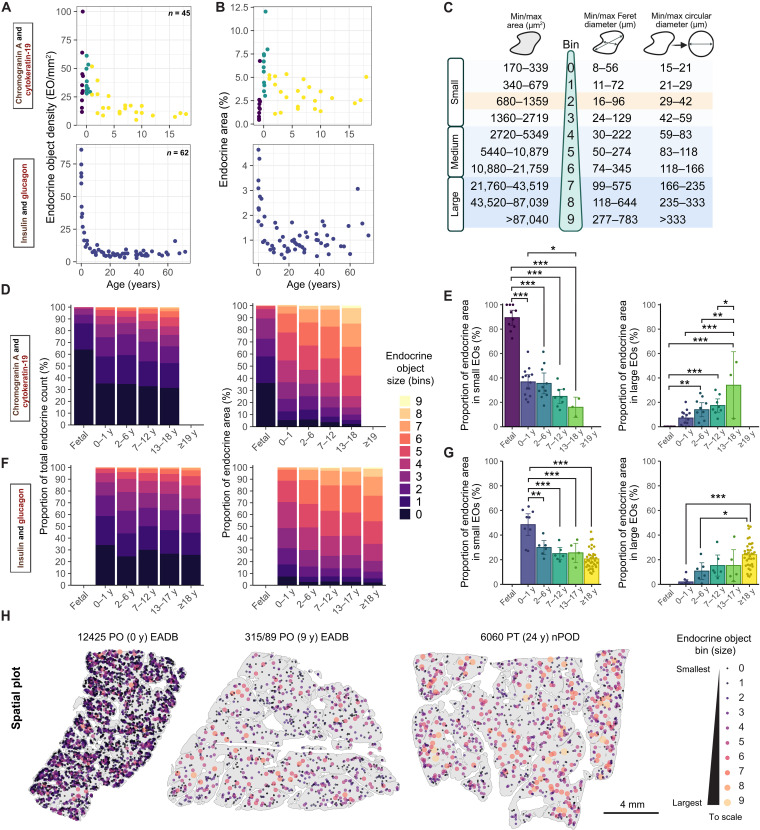
The endocrine mass in ND is contained within increasingly larger EOs with age, as the pancreas matures postbirth, which coincides with a reduction in EO density. (**A**) Changes in EO density with age in ND pancreata immunolabeled with CgA/CK19 (*n* = 45) or Ins/Gluc (*n* = 62). (**B**) Changes in the endocrine area (%) with age. (**C**) Schematic of EO area, bin, and diameter measurements. Single cells or contiguous clusters of endocrine cells are described as EOs. The EO area was log_2_ transformed to obtain the EO bin; bins 0 to 3 are designated small EOs, bins 4 to 6 are designated medium EOs, and bins 7 to 9 are designated large EOs. The approximate cutoff size for islets in many previous studies in the literature (*26**,* *66**,* *82*) is indicated in orange. (**D**) Proportion of total EO count and area in EO bins (0 to 9) in CgA/CK19-labeled ND pancreata. y, years. (**E**) Proportion of endocrine area in small EOs (bins 0 to 3) and large EOs (bins 7 to 9) across selected age brackets in CgA/CK19-labeled pancreata. (**F**) Proportion of total EO count and area in each EO bin (0 to 9) from Ins/Gluc-labeled ND pancreata. (**G**) Proportion of endocrine area in small EOs (bins 0 to 3) and large EOs (bins 7 to 9) across selected age brackets in Ins/Gluc-labeled ND pancreata. (**H**) Representative spatial plots of EO distribution and size in ND pancreata at birth (0), 9, and 24 years of age. Color and size of points indicate the EO bin size. Each section is labeled with donor ID, pancreas location [PH (pancreas head), PB (pancreas body), PT (pancreas tail), and PO (other/unknown)], age, and biobank. Data are presented as mean or mean and scatter ± 95% confidence interval (CI). Kruskal-Wallis test followed by Tukey’s post hoc test was performed to calculate *P* values. *P < 0.05, **P < 0.01, and ***P < 0.001.

Using donor tissue immunolabeled for CgA and CK19 (*n* = 45; HDBR *n* = 10, EADB *n* = 35), we quantified total EO density (Fig. 2A) and percentage of endocrine area (Fig. 2B) in donors without diabetes. A reduction in EO density and proportional endocrine area was apparent over the first 10 years of life and was particularly prominent during the first 2 years, coinciding with marked acinar expansion (Fig. 2, A and B, and fig. S1A). The EO area data were transformed to generate bins as previously described (*17*) and were further grouped into small, medium, and large sizes (Fig. 2C and fig. S2A). EO bin 0 equates to an area of 170 to 399 μm^2^ (approximately 1 to 3 cells), whereas bin 6 equates to an area of 10,880 to 21,579 μm^2^ and a diameter of 122 to 180 μm, approximately the size of an islet equivalent (150 μm^2^) (*21*). Visualization of AI-generated annotations and quality control (QC) comparisons of pipeline outputs on serially labeled CgA and Ins/Gluc sections confirmed that analysis pipelines were robust and reproducible (fig. S2B).

A total of 42,793 EOs was identified across the 45 donor sections examined. The frequency of EOs in each bin, and the contribution of each bin to overall endocrine area, was assessed across donor age groups (fetal, 0 to 1, 2 to 6, 7 to 12, and 13 to 18 years). In all age groups, most (76.3 to 98.6%) of the EOs identified were small and contributed to a minor proportion of the total endocrine area (15.9 to 36.7%; Fig. 2, D and E, and table S3). There was, however, a clear shift with age toward a greater frequency of large EOs that occupy an increasing proportion of the overall endocrine area (Fig. 2, D and E). This shift was confirmed in a second cohort of ND donors (*n* = 62; nPOD = 36, EADB = 20, and EUnPOD = 6) aged 0 to 72 years, where β and α cells were visualized using Ins and Gluc immunolabeling (Fig. 2, F and G). A total of 108,596 EOs was identified across donors from different age groups (0 to 1, 2 to 6, 7 to 12, 13 to 17, and ≥18 years). The EO profiles in age-matched donors from contemporary organ donor (nPOD and EUnPOD), live donor (DiViD), and archival (EADB) biobanks were concordant (fig. S2C). Conversely, EO density reduced with age across the different bin sizes; the greatest density of small and medium EOs was observed in the youngest donors (Fig. 2A). Age-dependent architectural changes are visually apparent in representative spatial plots of EOs, which are proportionally sized and colored according to bin size (Fig. 2H and fig. S3A). These plots illustrate the pronounced increase in large EO density and the corresponding reduction in overall EO density with age.

### Classification of EOs based on hormone content reveals that most small clusters comprise exclusively β cells

In their recently published 3D analysis of the whole human adult pancreas, Lehrstrand *et al.* (*15*) separated EOs based on the presence of Ins alone (β cells only; Ins^+^Gluc^–^), Ins and Gluc (β and α cells; Ins^+^Gluc^+^), and Gluc alone (α cells only; Ins^–^Gluc^+^). In the present analysis, the Ins- and Gluc-positive areas within each distinct EO, from individuals with T1D (*n* = 114 donors; 89,329 EOs) and ND individuals (*n* = 62; 108,596 EOs), were identified. EOs were classified on the basis of their endocrine content (Ins^+^Gluc^–^, Ins^+^Gluc^+^, and Ins^–^Gluc^+^); positivity for each hormone was determined as a labeled area greater than 40 μm^2^ (fig. S4, A and B).

**Fig. 3.**
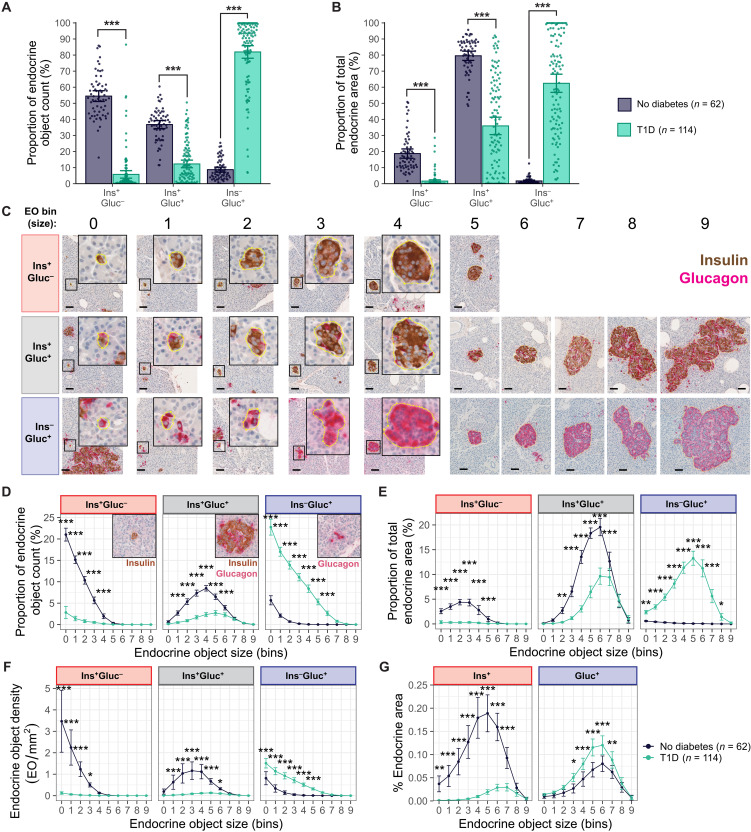
Small Ins^+^-only EOs are virtually absent in T1D, whereas β cells in larger objects persist. (**A** and **B**) Proportion of total EO count (A) and area (B) in EOs classified by their endocrine content (Ins^+^Gluc^–^, Ins^+^Gluc^+^, and Ins^–^Gluc^+^) in pancreata from donors with or without T1D. Mann-Whitney *U* test with Benjamini-Hochberg correction performed to calculate adjusted *P* values. Data are mean and scatter ± 95% CI. (**C**) Example bright-field images of EOs across bins, containing either Ins^+^ cells and/or Gluc^+^ cells. Scale bars, 50 μm. (**D** and **E**) Proportion of total endocrine count (D) and area (E) in each EO bin, classified by endocrine content. (**F**) EO density in bins, classified by endocrine content, in pancreata from donors with or without T1D. (**G**) Percentage of Ins- or Gluc-labeled area as a proportion of the tissue area in each EO bin. Type II analysis of variance (ANOVA) followed by Tukey post hoc comparing ND and T1D for each bin was performed to calculate *P* values. Data are presented as mean ± 95% CI. *P < 0.05, **P < 0.01, and ***P < 0.001.

Ins^+^Gluc^–^ EOs were most frequent in donors without diabetes (Ins^+^Gluc^–^: 54.5 ± 3.4%, Ins^+^Gluc^+^: 36.8 ± 2.5%, and Ins^–^Gluc^+^: 8.7 ± 1.6%; Fig. 3A) but represented a smaller proportion of endocrine area than Ins^+^Gluc^+^ EOs (Ins^+^Gluc^–^: 18.7 ± 2.9%, Ins^+^Gluc^+^: 79.5 ± 2.9%; and Ins^–^Gluc^+^: 1.7 ± 0.5%; Fig. 3B), indicating a likelihood that they are smaller in size. To assess size, we segregated EOs into EO bins (Fig. 2C) and classified them on the basis of their endocrine content. Representative examples are shown in Fig. 3C. Binning EOs revealed that 96% of Ins^+^Gluc^–^ are small, and 99.1% of Ins^–^Gluc^+^ are small, whereas Ins^+^Gluc^+^ EOs tend to be medium or large (Fig. 3D and fig. S3B).

### Extraislet β cells are virtually absent in T1D

A comparable analysis in donors with T1D identified a smaller total proportion of Ins^+^Gluc^–^ EOs compared to donors without diabetes (ND: 54.5 ± 3.4% and T1D: 5.8 ± 2.3%; *P* < 0.001; Fig. 3A). Ins^+^Gluc^–^ EOs contributed to a smaller proportion of overall endocrine area (ND: 18.7 ± 2.9% and T1D: 1.6 ± 0.8%; *P* < 0.001; Fig. 3B). The Ins^+^Gluc^+^ EOs also made up a smaller proportion of total EOs in individuals with T1D (ND: 36.8 ± 2.5% and T1D: 12.3 ± 2.4%; *P* < 0.001; Fig. 3A), and their corresponding area was smaller (ND: 79.5 ± 2.88% and T1D: 35.9 ± 5.4%; *P* < 0.001; Fig. 3B). Conversely, Ins^–^Gluc^+^ EOs were more numerous (ND: 8.7 ± 1.6% and T1D: 81.9 ± 3.9%; *P* < 0.001; Fig. 3A) and contributed more to overall endocrine area (ND: 1.7 ± 0.5% and T1D: 62.4 ± 5.7%; *P* < 0.001; Fig. 3B).

Examination of EO sizes classified by their endocrine content confirmed that small Ins^+^Gluc^–^ EOs were most affected in T1D with lower overall frequency, contribution to endocrine area, and density compared to ND (Fig. 3, D to F). The residual Ins^+^Gluc^+^ EOs in the T1D donors were medium and large (Fig. 3E). In T1D, the contribution of Ins^–^Gluc^+^ EOs to endocrine area was significantly greater than in donors without diabetes (*P* < 0.001; Fig. 3E), confirming that residual EOs retain α cells in the absence of β cells. A large proportion of these Ins^–^Gluc^+^ EOs were small (Fig. 3D), indicating more single α cells and small clusters of α cells, which are not frequently observed in donors without diabetes.

Inspection of total Ins^+^ and Gluc^+^ endocrine areas as a proportion of tissue area in T1D confirmed the significant loss of Ins^+^ cells across the first seven EO bins (Fig. 3G). Consistent with the lower frequency of the small β cell–only EOs and the trend toward larger residual Ins^+^Gluc^+^ EOs in donors with T1D, the residual Ins^+^ area was predominantly confined to EO bins 4 to 9. Assessment of further morphological measurements of EOs determined that EOs were less circular and solid with increasing size; this was more pronounced across bins 3 to 7 in donors with T1D compared to donors without diabetes (fig. S5, A and B).

### Earlier diagnosis of T1D correlates with increased loss of β cells in medium and large EOs

**Fig. 4.**
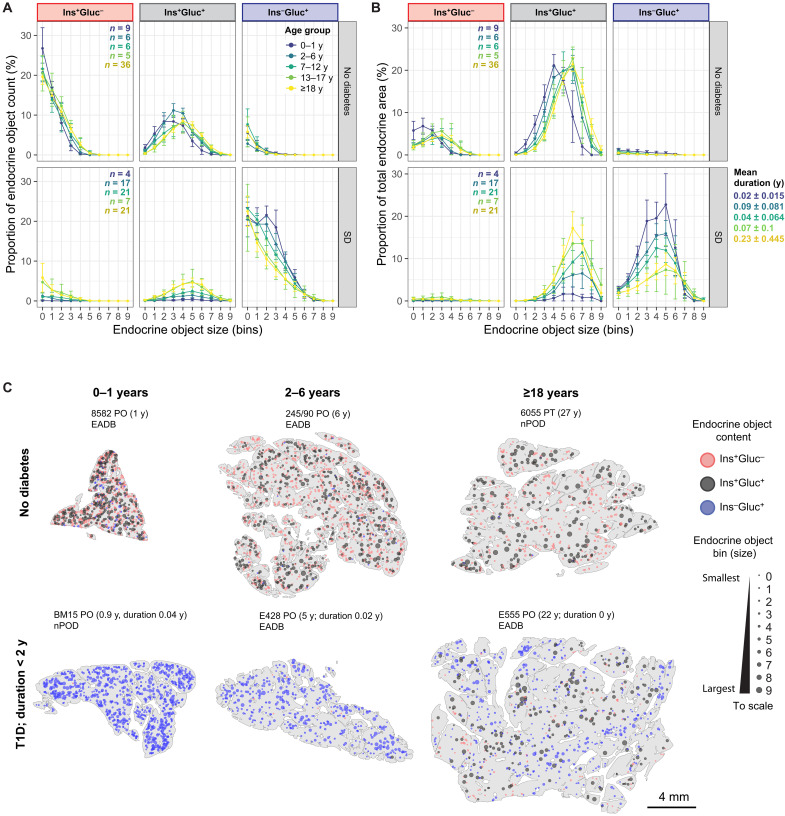
β cells contained within larger EOs, which are more prevalent in older individuals, are more likely to persist in short-duration (<2 years) T1D. Proportion of total EO count (**A**) and area (**B**) in each bin, classified by endocrine content, in donors with or without T1D (duration < 2 years), grouped by age at demise. (**C**) Representative spatial plots of pancreas sections from donors with or without T1D (duration < 2 years) including neonates, children, and adults. Each EO is represented by a single point; size and color correspond to the EO bin and the EO content, respectively. Each section is labeled with donor ID, pancreas location (PH, PB, PT, and PO), age, biobank, and duration of T1D. Data are presented as mean ± 95% CI.

We previously characterized two pancreatic endotypes of T1D, which are closely correlated with age at clinical diagnosis (*3*, *4*). Diagnosis under the age of 7 years is associated with the most intense immune infiltration and a lower frequency of residual Ins-containing islets at onset [T1D endotype 1 (T1DE1)] when compared with individuals diagnosed later in life [≥13 years; T1D endotype 2 (T1DE2)] (*3*, *4*). To explore the impact of age at T1D diagnosis on EOs, we assessed EO parameters in donors with a short duration of T1D (<2 years; *n* = 70) and compared the outcomes with age-matched donors without diabetes (Fig. 4). Donors were separated by age group to account for previously determined age-related architectural changes and allow interrogation of the impact of disease. A clear age-dependent gradient in EO parameters was observed, where the individuals diagnosed at younger ages exhibited an increasingly and incrementally severe loss of Ins^+^Gluc^+^ EOs, measured by proportion of total EO count (Fig. 4A) and proportion of total endocrine area (Fig. 4B). The virtual absence of Ins^+^Gluc^–^ EOs was apparent in all short duration T1D donors regardless of age at onset, with both count and area reduced (Fig. 4, A and B). A shift toward a progressive retention of larger Ins^+^Gluc^+^ EOs was observed in T1D donors with increasing age when compared with their age-matched controls. The Ins^–^Gluc^+^ proportional area correspondingly reduced in the T1D donors with increasing age. The maturation of endocrine architecture with age is a driving factor for changes in EO density and percentage of endocrine area and emphasizes the importance of comparing age-matched samples for donors with and without T1D (fig. S6). Spatial plots visually represent these observed changes and highlight the lobularity of disease, which is frequently reported (Fig. 4C). These findings support the conclusion that β cell loss is more profound in individuals diagnosed with T1D at an early age compared with those diagnosed at an older age.

### EOs are not affected in single AAb^+^ donors without diabetes

**Fig. 5.**
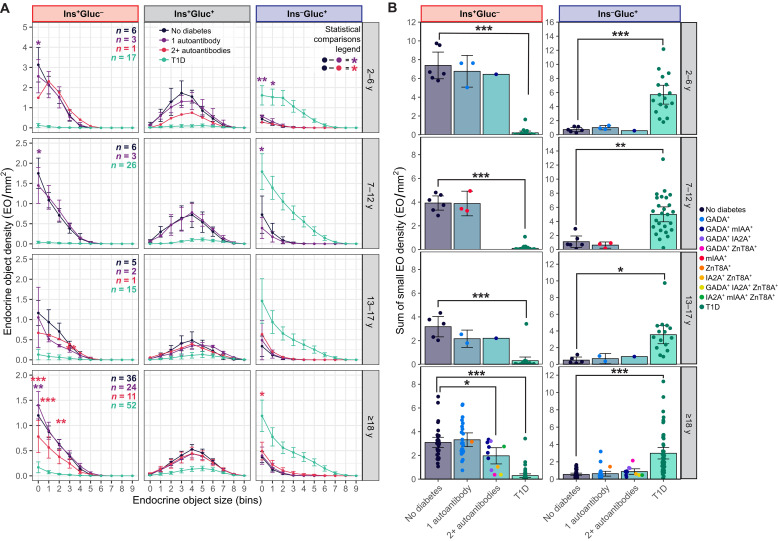
The presence of a single AAb is not associated with a significant loss in small Ins^–^Gluc^+^ EOs overall, but multiple AAb^+^ donors aged ≥18 years do exhibit a reduction in small Ins^–^Gluc^+^ EOs. (**A**) EO density in bins classified by endocrine content in age-matched donors without diabetes, single AAb^+^, multiple AAb^+^, and donors with T1D. Type II ANOVA with Dunnett’s post hoc test comparing single and double AAb^+^ donors to donors without diabetes was performed, where *n* ≥ 3, to calculate *P* values for each EO bin. (**B**) The sum of small Ins^+^Gluc^–^ EO density (bins 0 to 3). The data point color indicates detected AAbs. Type II ANOVA with Dunnett’s post hoc test comparing single and double AAb^+^ donors and donors with and without T1D was performed, where *n* ≥ 3, to calculate *P* values for each EO bin. Data are presented as mean and scatter ± 95% CI. *P < 0.05, **P < 0.01, and ***P < 0.001.

We demonstrated changes in the EO area, density, and morphology consistent with age and progression of T1D. However, to determine whether T1D-associated alterations are due to disease itself, rather than a predisposing factor such as a developmental impairment, we assessed relevant parameters in 45 AAb^+^ donors without diabetes. Initially, donors were grouped according to the number of AAbs detected [single AAb^+^ (*n* = 32) or multiple AAb^+^ (*n* = 13)] and their age (Fig. 5A).

Young donors with a single AAb had minimal alterations in EO density compared to donors without diabetes, with any significant changes present only in the smallest bin sizes (0 and 1) (Fig. 5A). Notably, adult donors (≥18 years) with a single AAb had a greater density of the smallest Ins^+^Gluc^–^ EOs compared to donors without diabetes (Fig. 5A). In contrast, adult donors (≥18 years) with two or more AAb had a lower density of small Ins^+^Gluc^–^ EOs compared to donors without diabetes (Fig. 5, A and B) and a greater density of the smallest Ins^–^Gluc^+^ EOs (Fig. 5A). Examination of the small EO density of donors categorized on the basis of their AAb type {glutamic acid decarboxylase 65 (GADA), Insulin [microinsulin antibody assay (mIAA)], insulinoma-associated antigen 2 (IA2A), or zinc transporter 8 (ZnT8A); Fig. 5B} revealed that adult donors with IA2A^+^ and one or more AAb trended toward a lower density of Ins^+^Gluc^–^ EOs and a higher density of Ins^–^Gluc^+^ EOs (Fig. 5B). Conversely, adult multiple AAb donors with GADA had a comparable density of small Ins^+^Gluc^–^ EOs to donors without diabetes. Further breakdown of impacts on EO characteristics is found in fig. S7 (A to D).

### EO parameters are affected in AAb^+^ donors without diabetes that have additional features associated with progression toward clinical T1D

Islet AAb positivity alone is not sufficient to predict whether an individual will progress to overt clinical disease. Most donors with islet AAbs, particularly a single AAb, do not (*22*). However, emerging evidence suggests that AAb^+^ individuals with IA2A^+^ are at greater risk of progressing toward clinical diabetes (*23*). The T1D staging criteria predict progression using the presence of multiple AAbs, combined with measures of glucose control (*24*, *25*), but other histological hallmark features of T1D can be useful for predicting progression in donors where the pancreas is available, such as the presence of insulitis and hyperexpression of human lymphocyte antigen class I (HLA-I) (*26*–*28*). Last, a higher T1D genetic risk score (T1D-GRS) (*29*–*31*) in AAb^+^ donors is also associated with progression to T1D.

**Fig. 6.**
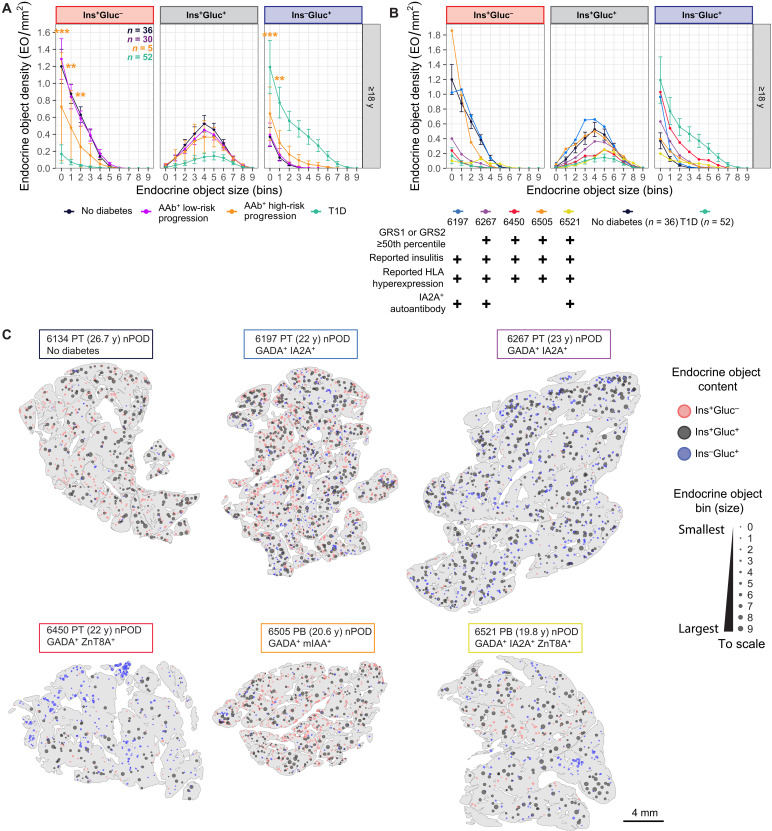
A subset of donors with multiple indicators of T1D progression, including IA2A^+^, high T1D-GRS, and histological features of T1D, have a reduced density of Ins^+^Gluc^–^ EOs and a greater density of Ins^–^Gluc^+^ EOs. (**A**) EO density in each bin, separated by endocrine content, in age-matched donor pancreata, grouped by the presence of IA2A and numbers of AAbs. Type II ANOVA with Dunnett’s post hoc test comparing single and double AAb^+^ donors to antibody-negative donors without diabetes was performed to calculate *P* values for each bin. (**B** and **C**) EO density in bins separated by endocrine content (B), and spatial plots (C) comparing IA2A^+^ donors to age-matched donors with or without T1D. Clinical data used to designate risk can be found in table S1. Data are presented as mean ± 95% CI. *P < 0.05, **P < 0.01, and ***P < 0.001.

We identified donors at greater risk of progression according to whether they met at least three of the following criteria: GRS1 or GRS2 ≥ 50th percentile [available via the nPOD Data Portal (*29*–*31*)], reported insulitis (nPOD Data Portal), HLA-I hyperexpression (*27*, *28*, *32*), or IA2A^+^. Five multiple AAb^+^ donors met this threshold (table S1), and the rest were designated as low risk. These donors showed a significantly lower density of small Ins^+^Gluc^–^ EOs compared to donors without diabetes and a significantly greater density of small Ins^–^Gluc^+^ EOs (Fig. 6A). A full comparison of measured EO metrics is included in fig. S9 (A to D). On an individual basis, three of the five high-risk AAb^+^ donors (nPOD 6267, 6450, and 6521) more closely resembled the donors with T1D (Fig. 6B and fig. S10, A to D). All three donors had a high T1D-GRS and reported insulitis, with two donors meeting all four criteria. Representative spatial plots of the high-risk AAb^+^ donors and a similarly aged donor without diabetes visually demonstrate the lack of small Ins^+^Gluc^–^ EOs and increased levels of small and medium Ins^–^Gluc^+^ EOs, specifically in these three donors (Fig. 6C). Low-risk AAb^+^ donors displayed similar hemoglobin A1c (HbA1c) (%) and C-peptide levels to age-matched donors without diabetes (fig. S8, A and C). Notably, high-risk donors also had HbA1c levels within a healthy range but slightly elevated C-peptide levels compared to donors without diabetes, which may suggest that histological changes precede alterations in glucose control measures in these donors. In summary, we observe alterations in EO parameters that resemble changes observed in individuals with short T1D duration in AAb^+^ donors with multiple indicators of progression toward T1D, which may be associated with pancreatic autoimmunity.

### There is a lower density of large islets in individuals diagnosed with T1D at an early age (<13 years)

**Fig. 7.**
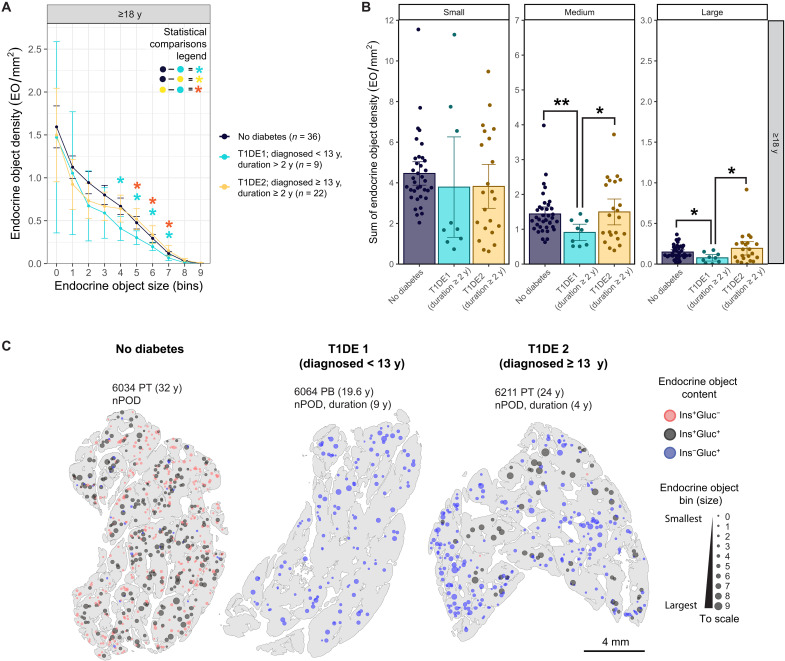
Clinical onset of T1D < 13 years interrupts the pathway for the generation of larger EOs. (**A** to **C**) EO density in bins (A); sum of small, medium, and large EO density (B); and representative spatial plots (C) of adult ND, T1DE1 (diagnosed < 13 years), and T1DE2 (diagnosed ≥ 13 years) pancreata with a disease duration of ≥2 years. Mann-Whitney *U* test and Benjamini-Hochberg procedure were used to calculate adjusted *P* values. Data are presented as mean and scatter ± 95% CI. *P < 0.05, **P < 0.01, and ***P < 0.001.

Throughout these analyses, we identified multiple small β cell–only EOs present at a high frequency and density in young, healthy individuals, which are virtually absent in select multiple AAb^+^ donors and in individuals recently diagnosed with T1D, particularly those with a young onset of disease. We also show that the youngest donors have the smallest EO size profile, which then gives way to larger EOs with age. It is well established from natural history studies that individuals who develop islet AAbs at a very young age (<2 years) are at greater risk of progression to clinical diabetes than those who develop AAb later in childhood (*33*–*35*). Therefore, to examine whether earlier initiation of islet autoimmunity, when the pancreas has a high density of small and potentially more vulnerable EOs, has a more profound impact on the formation of larger EOs later in life, we studied adult donors with T1D with a longer duration of disease (≥2 years), diagnosed either < 13 years (mainly T1DE1) or ≥ 13 years (T1DE2) based on our previously defined T1D pancreatic endotypes. We assessed the density of all EOs, independent of endocrine content (Fig. 7A). No significant difference in small EOs was apparent, likely due to the lack of small β cell–rich EOs and an increase in small α cell–rich EOs, which had lost β cell mass, in T1D donors, as reported earlier. However, the density of medium and large EOs was significantly lower in individuals diagnosed <13 years compared to both the donors without diabetes and the donors diagnosed ≥13 years (Fig. 7B). Representative spatial plots confirm these differences (Fig. 7C). Together, these results suggest that early onset of T1D may interrupt the pathway for the generation of larger EOs.

### Residual Ins^+^ area is greater in individuals who developed clinical T1D at a later age (≥13 years), and this is confined to larger EOs

**Fig. 8.**
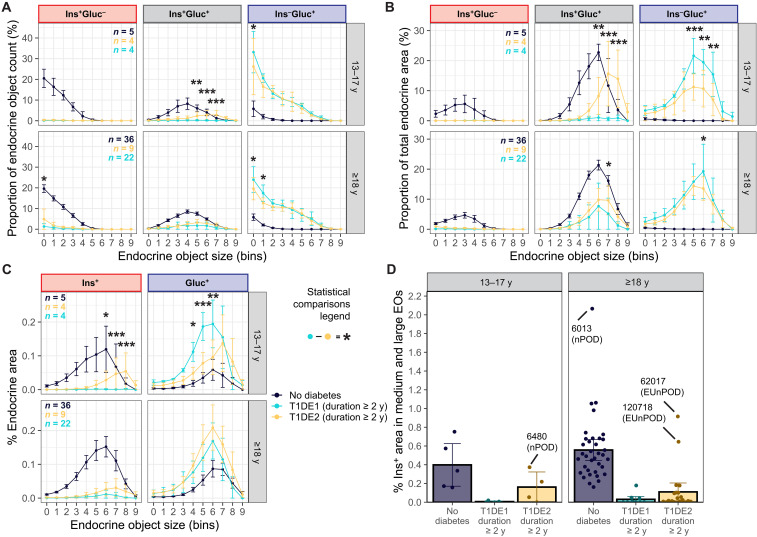
Clinical onset of T1D ≥ 13 years is associated with preservation of β cells in larger EOs in a subset of T1D cases with a duration of ≥2 years. (**A** and **B**) Proportion of EO count (B), area (**C**), and hormone^+^ area as a proportion of the tissue area in each EO bin classified by endocrine content for age-matched ND, T1DE1 (duration ≥ 2 years), and T1DE2 (duration ≥ 2 years) pancreata. Type II ANOVA with Tukey’s post hoc test comparing T1DE1 to T1DE2 for each bin, separated by endocrine content, was performed to calculate *P* values. (**D**) Sum of the percentage of Ins^+^ area contained within medium and large EOs. Outliers are labeled by donor ID. The Mann-Whitney *U* test was performed to calculate *P* values. Data are presented as mean and scatter ± 95% CI. *P < 0.05, **P < 0.01, and ***P < 0.001.

Previously, clinical onset of T1D at an early age (<13 years) was associated with marked loss of β cell mass (*3*, *4*, *36*). We next investigated whether the age of diagnosis affects the EO size profile and content in age-matched T1D donors with a duration of ≥2 years. We opted to define T1DE1 in this study as an age of diagnosis < 13 years because these age groups shared a similar reduction in Ins^+^Gluc^+^ EOs as a proportion of total count in T1D (disease duration < 2 years) compared to donors without diabetes (Fig. 4A and fig. S6A). T1DE2 was defined by age of diagnosis ≥ 13 years. Following categorization of donors as T1DE1 or T1DE2, we explored EO profiles based on age at demise, separating them into groups (13 to 17 and ≥18 years). In the 13- to 17-year group, T1DE1 individuals had a smaller percentage of large Ins^+^Gluc^+^ EOs and a greater percentage of the smallest Ins^–^Gluc^+^ EOs compared to individuals diagnosed ≥ 13 years (Fig. 8A). Large Ins^+^Gluc^+^ EOs also contributed less to the overall endocrine area in T1DE1s versus T1DE2s and large Ins^–^Gluc^+^ more so (Fig. 8B). In the ≥18-year group, T1DE1 individuals had a lower proportion of the smallest Ins^+^Gluc^–^ EOs and a greater proportion of small Ins^–^Gluc^+^ EOs (Fig. 8A). When considering the percentage of endocrine area of the pancreas section attributed to Ins and Gluc, respectively, a significantly smaller Ins-positive area was represented in large islets in T1DE1 individuals versus T1DE2 individuals for ages 13 to 17 years, suggesting that large EOs have greater numbers of β cells (Fig. 8C). A similar trend was observed for the ≥18-year age group but was not significant. The sum of the percentage of Ins^+^ area in medium and large bins shows minimal β cell mass remains in T1DE1 donors relative to the pancreas tissue area (Fig. 8D and fig. S11). In contrast, a subset of T1DE2 donors showed greater preservation of β cell mass in medium to large EOs, although some had equivalent relative β cell mass to the T1DE1 donors, indicating greater heterogeneity in the T1DE2 donor group (fig. S12). Comparisons of all EO measurements assessed for T1DE1 and T1DE2 donors with a duration of <2 and ≥2 years can be found in fig. S13 (A to D), and comparisons of HbA1C and C-peptide levels can be found in fig. S8 (B and D).

## DISCUSSION

The recent 3D visualization of an entire human pancreas (*15*) emphasized the presence of small hormone-positive cell clusters located remotely from the more classically defined islets of Langerhans. The notable number of these small “EOs” was largely, although not totally (*16*–*20*), unappreciated in earlier studies. This may reflect the heavy reliance on 2D analysis of pancreatic tissue, in which clusters of cells cannot be readily assigned as “extraislet” structures due to the topography of the tissue. In contrast, mesoscopic optical 3D imaging approaches provide unequivocal evidence that as many as 50% of the EOs are smaller than classical islets and consist primarily of Ins^+^ β cells, with few or no Gluc^+^ α cells (*15*). The present study builds on these newer 3D (*15*, *37*, *38*) and early 2D (*16*–*20*, *39*) studies by applying an innovative analysis pipeline to define the endocrine cell topography in 2D pancreas sections from 250 donors across a wide range of ages, held in multiple biobanks. We confirm that among ND individuals, 40 to 60% of total EOs exist as small clusters of cells predominantly comprising β cells. We further demonstrate that the relative endocrine area (as a proportion of total pancreas area) and the EO density each reduce consistently in the first few years of life, with endocrine area stabilizing at 1 to 2% of total pancreas area by the early teenage years. Both an earlier (*20*) and two very recent studies (*40*, *41*) using different pancreas biobanks reached similar conclusions.

Postmortem studies of pancreas weight at increasing ages support the notion that the pancreas expands until the age of ~30 years (*42*–*44*). Maintenance of endocrine area at 1 to 2% of the pancreas beyond the teenage years implies that endocrine cell expansion continues in some form over at least the first three decades of life. Debate as to whether this sustained increase in endocrine mass, especially beyond the age of 10 years, is derived from endocrine proliferation or neogenesis is ongoing (*20*, *45*–*49*). While the present study cannot provide a resolution, applying our methods in future studies focused on the proliferation status of the smaller EOs and/or their proximity to ductal structures, as potential sites of endocrine neogenesis, should provide valuable new insights.

Our analysis of thousands of pancreatic EOs throughout early life and into adulthood firmly demonstrates that the proportion of the EO area allocated within the smallest EO bins is greatest in early childhood and that a pronounced shift toward larger EOs occurs with increasing age. This change coincides with a period of rapid pancreatic growth and extensive architectural changes largely overlooked in earlier work. The lack of a thorough assessment of these changes, and the paucity of information about the likely functional significance of the small EOs, given their loss during islet isolation, leaves many questions outstanding. These include the conundrum that these small EOs may be distinct entities with specific functions that differ from those attributed to a typical islet and that their functionality and topography may change throughout the life course. It is tempting to speculate that the shift toward larger EOs with age is evidence of a neogenesis pipeline culminating in larger EO (islet) generation, with EOs at different stages of this developmental process present within any particular individual (*50*–*52*). Given that studies of pancreas tissue and isolated islets demonstrate enhanced insulin secretion, greater insulin content, a higher proportion of β cells, and a greater percentage of β cells in direct contact with blood vessels in smaller islets, it is likely that EO size and composition are functionally important (*50*, *52*, *53*).

Arguably, this study’s most notable and significant observation is the virtual absence of small EOs in the pancreas of donors with T1D. This implies that the smaller, β cell–rich objects may be particularly susceptible to autoimmune-mediated destruction. These observations align with 3D imaging studies in the nonobese, autoimmune mouse model, where smaller islets are also least resistant to immune attack (*54*). These findings have been very recently confirmed in both 3D (*38*) and 2D (*41*, *55*) assessments in different cohorts. In contrast, in the streptozotocin (STZ)–induced model of diabetes, where β cell toxicity rather than autoimmunity causes β cell loss, the small EOs are preserved, with the larger islets affected (*56*). This suggests a functional difference between the β cells in small clusters versus classical islets, at least in mice, because STZ requires the expression of Glucose Transporter Type 2 (GLUT2) for its uptake, which may be lacking in extraislet β cells. However, the impact of STZ on endothelium and its consequential impact on small EOs cannot be ruled out (*57*, *58*). Furthermore, despite our inability to examine in detail immune infiltration around small EOs in human T1D pancreas, due to their virtual absence, the evidence of elevated acinar inflammation in individuals with T1D, and the presence of islet-reactive CD8^+^ T cells in the exocrine compartment, supports the presence of an immune component capable of targeting β cells in the extraislet environment (*59*–*61*). This could reflect remnants of active β cell autoimmunity and aligns with the persistence of Ins^–^Gluc^+^ small EOs. Consistent with this hypothesis, residual β cells were predominantly confined to larger EOs, particularly in donors clinically diagnosed at the youngest ages. This suggests that β cells contained within the large EOs may be initially protected from autoimmune-mediated destruction and that smaller EOs are lost early in the disease process, supported by a lack of the smallest Ins^+^Gluc^–^ EOs in select individuals with multiple islet AAbs and other indicators of progression. Factors such as the presence of an intact islet basement membrane (reducing accessibility of immune cells), the localization and number of protective accessory cells (e.g., pericytes and mesenchymal stromal cells), and/or the β cells mounting an active repulsive response could provide an explanation (*32*, *62*–*65*).

We also observed a lower EO density in individuals with T1D compared to age-matched donors without diabetes. This aligns with an earlier study undertaken in a limited number of T1D donors diagnosed in adulthood (*66*). This lower density was particularly apparent for medium to large EOs in children who developed diabetes at <13 years versus age-matched ND donors. This implies that the selective targeting of small EOs early in the autoimmune process could negatively affect the generation of larger EOs during childhood, thereby resulting in a reduced density of all EOs in later life. Hence we propose that, particularly in young children, T1D may be a disease of β cell loss [driven by a more aggressive immune-mediated destruction (*3*, *4*)] coupled with a net initial β cell deficit, resulting from a failure to establish larger EOs in sufficient quantities. In individuals diagnosed in their teens and beyond, β cells are preferentially preserved in the medium and large EOs for extended periods postdiagnosis, perhaps as these are formed before initiation of the autoimmune attack. These findings align with clinical observations showing that individuals diagnosed beyond their earliest years are more likely to retain the ability to make at least some endogenous insulin (assessed by C-peptide microsecretion) (*36*, *67*) and exhibit less florid insulitis (*4*, *68*). Nevertheless, cross-sectional studies measuring C-peptide decline with increasing disease duration support a continuous loss of residual functional β cells (at least for the first 7 years) (*67*). Notably, when comparing the size of the Ins^–^ and Ins^+^ EOs, the shift in bin size with age was less pronounced in individuals diagnosed ≥ 13 years than in individuals diagnosed < 13 years. This corresponds with emerging studies suggesting that the dedifferentiation or transdifferentiation of β cells under conditions of prolonged metabolic stress could reduce the β cell area without causing a substantial loss of endocrine area [reviewed in (*69*, *70*)]. It is tempting to speculate that a loss, or lower initial proportion, of smaller EOs sitting at an early point in the proposed EO renewal pipeline would put an additional metabolic strain on the residual β cells within larger EOs, driving pathological outcomes such as β cell dedifferentiation, transdifferentiation, senescence, and β cell stress (*71*–*74*). Studies demonstrating that excess body mass index in childhood is associated with faster progression toward clinical disease are consistent with this concept (*75*, *76*). Last, although limited by sample size, the data suggest that most donors without diabetes who are positive for a single AAb, and many of the donors with multiple AAb, had no significant reduction in small Ins^+^Gluc^–^ EOs when compared to age-matched donors without diabetes, although there were some reductions in the very smallest (bin 0) in the youngest donors. This finding was confirmed in two very recent studies (*38*, *41*). This reduces the possibility that a defect in early development in at-risk individuals accounts for the alterations in EO frequency and density observed in clinical disease. In contrast, those with pathological indicators of T1D progression [presence of IA2A AAb, insulitis, HLA hyperexpression, or a high T1D-GRS (*23*, *26*–*32*, *61*)] support our proposal that the small EOs are most susceptible to immune-mediated destruction.

Our collective observations have several potential implications for the understanding of normal pancreas development and T1D pathogenesis. Most notably, the data support the idea that β cell mass expansion continues within a critical time window postnatally. During this period, a transition occurs from smaller to larger EOs, with a greater proportion of the β cell mass residing in the medium to large EOs by 7 years of age. In contrast, the very–early-life period where the greatest pancreas expansion occurs correlates with the time when many β cells exist as single cells or in small clusters. It is currently unclear whether the rate of insulin secretion from these objects differs from larger EOs at any given glucose concentration. However, the fact that small EOs are preserved after STZ exposure in rodents, which may reflect reduced GLUT2 expression [see above (*56*)], is consistent with such a possibility. Furthermore, the lack of paracrine signals from neighboring endocrine cells and the incomplete architecture of the vasculature and innervation will likely also lead to differential responsiveness. One possibility is that these β cells secrete Ins continuously rather than in response to varying glucose concentrations, thereby acting as a trophic source for pancreatic expansion. Therefore, if these small Ins^+^ EOs are disrupted (e.g., through priming autoimmunity or via β cell stressors), their loss will strongly affect both endocrine mass expansion and pancreas growth. This fits with the observed reduction in pancreas weight in individuals with T1D and in those with specific monogenic forms of diabetes caused by enhanced β cell stress (*43*, *77*).

**Fig. 9.**
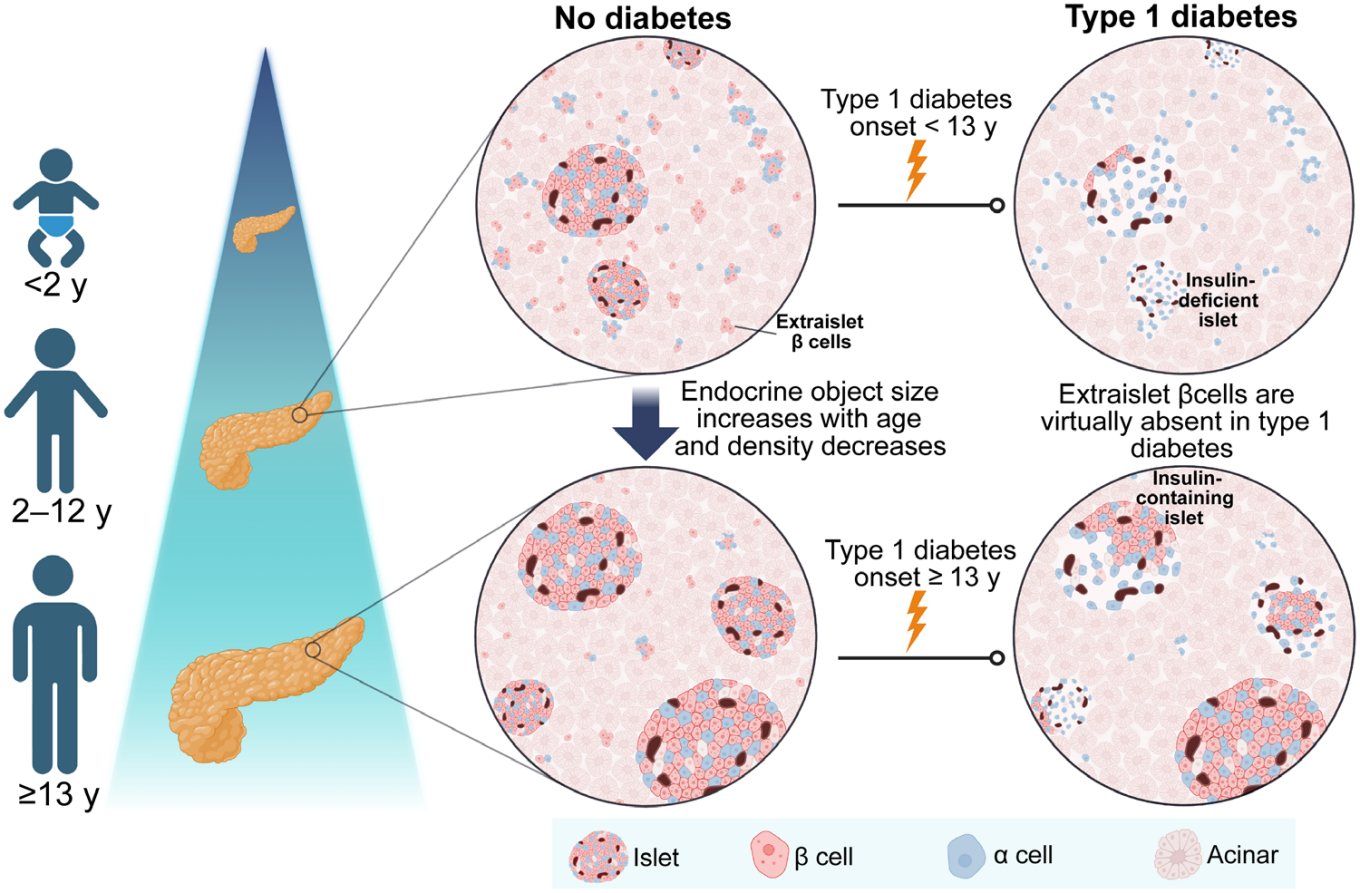
Summary of study findings. The endocrine area comprises mostly small, extraislet EOs in early life, with larger islets developing with age. Extraislet β cells are virtually absent in T1D, and islets retaining β cells are larger. Development of T1D in early life, when EOs are small, is associated with fewer large islets in adulthood. Lack, or early destruction, of extraislet β cells may therefore detrimentally affect larger EO generation.

In summary, we document profound changes in the architecture of the pancreas, particularly in the density and area of EOs in early life (Fig. 9). We confirmed and extended recent 3D studies reporting the presence of single cells and small clusters of β cells within the pancreas, observing them in donors of all ages without T1D. We show that the single cells and small clusters of β cells are virtually absent in individuals with T1D. We demonstrate that the density of larger EOs is lower in individuals with T1D and that this is most evident in the children who were youngest at clinical diagnosis. We propose that an early and selective elimination of single β cells and small clusters of β cells in young individuals may reduce their ability to generate a full complement of endocrine mass during development, resulting in more aggressive clinical disease.

This altered perspective has important implications for current and future treatments and for efforts to implement screening for individuals at risk of T1D. Our findings imply that a major proportion of β cells are lost during the islet isolation process. We speculate that the loss of these small EOs during isolation could help explain why whole pancreas transplantation is more successful than isolated islet transplantation, which frequently requires multiple transplants to reverse T1D durably (*51*, *78*). These findings substantiate a need for early, targeted immunotherapy intervention in stage 1 in high-risk individuals that could prevent loss, or promote the development, of single β cells and clusters, allowing larger, better-protected islets to develop. Furthermore, this study provides support for islet or stem cell–derived islet replacement strategies, especially in individuals diagnosed with T1D at a young age, who may not be able to generate a full complement of endocrine mass; i.e., what was absent initially cannot be protected or recovered (at onset). Last, this has implications for future β cell regenerative therapies, which might selectively promote the formation of single β cells or small clusters that are targeted by active autoimmunity, so a combination with immunotherapeutic agents will be important to protect the newly formed cells.

### Limitations of the study

The present study is limited in several ways, most notably by its cross-sectional nature, precluding interpretations of “loss” or “reduction,” and the availability of donors from certain age/disease groups. Nonetheless, we report on more donors than any previous study of pancreas morphology, particularly those with T1D. A mixture of postmortem, organ-donor, and live-donor pancreas tissue was included for study, owing to the nature of the biobanks from which tissue was procured. However, we observed no discernible differences in outcomes from these when compared. We also supplemented the work using archival donor tissue immunolabeled for Ins and Gluc; hence, we cannot exclude the possibility of other endocrine cell subsets, including cells undergoing de- or transdifferentiation, which may exist within the small EOs, beyond α-and β cells. The nature of chromogen staining also precludes reliable identification of multihormone-positive cells. These will be the subject of ongoing studies in available tissue. Most of the AAb^+^ donors available were aged ≥ 18 years (35/45; 77%), and there is limited information available about rates of progression toward T1D in this age group. Last, we acknowledge that our quantification was undertaken in sections from a mixture of pancreatic regions and that variation between these is likely.

## MATERIALS AND METHODS

### Study participant details

Pancreas tissue from human organ donors with or without T1D was procured for the nPOD program at the University of Florida (RRID: SCR_014641; www.jdrfnpod.org) and processed in accordance with the established standard operating procedures of the nPOD/Organ Processing and Pathology Core (OPPC) as approved by the University of Florida Institutional Review Board (IRB201600029) with strict adherence to the guidelines of the United Network for Organ Sharing and federal regulations, with informed consent obtained from each donor’s legal representative. The EADB is held with ethical permission from the West of Scotland Research Ethics Committee (ref: 25/WS/0017; Integrated Research Application System (IRAS) project ID: 354341). Human fetal tissues were obtained from the HDBR. These samples were collected with appropriate maternal written informed consent and approval from the Newcastle and North Tyneside (Newcastle University) and London—Fulham [University College London (UCL)] Research National Health Service (NHS) Health Authority Joint Ethics Committees (Ref: 23/NE/0135; IRAS project ID: 330783). HDBR is regulated by the UK Human Tissue Authority (HTA; www.hta.gov.uk) and operates in accordance with the relevant HTA Codes of Practice. The DiViD study was approved by The Norwegian Governments Regional Ethics Committee (IRB 0000 1870). Written informed consent was obtained from all cases after oral and written information from the diabetologist and the surgeon separately. Three women and three men, 3 to 9 weeks after diagnosis, (aged 24 to 35 years) participated. Detailed clinical characteristics have been described (*79*). Organs for the Medical Research Council (MRC) QUOD Whole Pancreas Biobank were retrieved after informed and written donor family consent in compliance with the UK Human Tissue Act of 2004 under specific ethical approvals by the UK Human Research Authority (05/MRE09/48 and 16NE0230). EUnPOD: The studies involving human participants were reviewed and approved by the local ethics committee of the University of Pisa (Italy). Pancreata not suitable for organ transplantation were obtained with informed written consent by organ donors’ next of kin and processed with the approval of the local ethics committee (*80*).

### Pancreas tissue

Donor pancreata were obtained from the EADB, HDBR, nPOD, including a small subset of archival samples from EUnPOD, and the diabetes virus infection study (DiViD) biobanks. A total of 458 tissue sections from 250 unique donors was analyzed. All clinical donor information can be found in table S1. Information about the location in the pancreas from which sections were obtained and whether they were newly stained or obtained from an archive can be found in table S2. Pancreas sections (4 μm) from the EADB and the HDBR were labeled for a combination of CgA and CK19 [10 fetal donors (*81*) and 35 neonatal and pediatric donors]. An additional donor cohort was obtained from the EADB, nPOD, EUnPOD, and DiViD biobanks in the form of WSIs of previously labeled archived tissue, some of which was performed in-house; Ins/Gluc (*n* = 106) and Ins/Gluc/CD45 (*n* = 85).

### Staining of pancreas sections

Pancreas sections were dewaxed with either 0.5% iodine in xylene or Histo-Clear (National Diagnostics, USA) and rehydrated using decreasing concentrations of ethanols. Heat-induced epitope retrieval in citrate buffer (pH 6.0) was used to unmask antibody-binding epitopes. Sections were blocked in tris-buffered saline with 5% normal goat serum before the application of primary antibodies. A dual anti-mouse horseradish peroxidase (HRP) and anti-rabbit alkaline phosphatase (AP) secondary antibody was applied, and a chromogen detection kit was used for secondary antibody detection [HRP–diaminobenzidine (DAB) and AP–Warp Red; Biocare Medical, USA] in slides immunolabeled with either CgA/CK19 or Ins/Gluc. For sections immunolabeled with Ins, Gluc, and CD45, the chromogen detection reagents DAB, AP-Warp Red, and AP–Ferangi Blue chromogen were used. Sections were mounted using either EcoMount (Biocare Medical, USA) or DPX Mountant for histology (Merck, UK).

### Imaging and analysis of EO architecture

Labeled sections were digitized at ×20 magnification using a PhenoImager HT slide scanner (Akoya Biosciences, USA) or an Aperio CS2 slide scanner (Leica Biosystems Imaging Inc.). WSIs were imported into the HALO image analysis platform (Indica Labs, USA). User-created annotations were used to train the DenseNet V2–convoluted neural network to demarcate tissue compartments of interest (connective tissue, acinar, duct, single endocrine cells, and those within small clusters and islets). The real-time tuning feature was used to assess classifier performance across iterations. The model was trained until the cross-entropy, a measure of difference between the network classification and user-created annotations, reached its lower value and plateaued, as the neural network converged on a solution. Separate DenseNet V2 classifiers were trained for slides stained with CgA/CK19 and Ins/Gluc. An additional classifier was trained for slides stained for either Ins/Gluc/CD3 or Ins/Gluc/CD45. Classifiers were trained to differentiate between EOs and staining artefacts. The minimum threshold for objects to be identified by the classifiers was set to 170 μm^2^, the approximate size of an endocrine cell (*17*). Human verification and QC of AI-created annotations helped further improve the quality of annotations used in downstream analyses.

For the Ins/Gluc-labeled sections, the HALO 3.6 area quantification algorithm was applied to calculate the Ins and Gluc positively labeled area within each EO. User-defined RGB values designated staining and hematoxylin colors for deconvolution, and an optical density threshold set the intensity for positive staining. This methodology attributed pixel values to one of the user-defined RGB values, except in cases where the optical densities of overlapping chromogens were at their strongest. This may lead to an underestimation of dual hormone–positive cells. Performance of the area quantification algorithm was reviewed on a section-by-section basis, as the optical density varied among historically labeled sections and was optimized if necessary.

Individual EO data were imported into “R” for processing, data visualization, and statistical analysis. The transformation of EO area into the EO bins was performed using the formula

$$\text{EO}\;\text{bin}={\text{log}}_{2}\left[\frac{\text{EO}\;\text{area}\;{\mathrm{\mu m}}^{2}}{\;{\left(170\;\mathrm{\mu m}\right)}^{2}}\right]$$

The area of each EO was divided by 170 μm^2^ (*17*) to calculate an approximate cell number per EO, followed by log_2_ transformation. The final EO bin value was rounded down to the nearest integer. This size scale allows for fine detail of the smaller EOs and larger bin sizes for the lower number of larger EOs (*17*). EOs in sections stained for Ins/Gluc were excluded from downstream analyses if the Ins^+^ or Gluc^+^ area was <40 μm^2^ to filter out potential staining artifacts erroneously identified by the AI classifier (fig. S4, A and B). Individual EO data and acinar tissue area were passed forward to calculate the EO size profiles, EO density (in square millimeters), and the percentage of endocrine area and acinar area.

EO annotations created using HALO-AI classifiers were imported into QuPath v5.1 as .geojson annotation files for further analysis of morphological characteristics of EOs, as these features were not available within the HALO platform. Annotations imported into QuPath do not share the same ObjectIDs as those in the HALO results table and need to be linked to each other. Therefore, each annotation in QuPath was mapped to an EO (Ins^+^Gluc^–^, Ins^+^Gluc^+^, and Ins^–^Gluc^+^) in the HALO result table based on the sum distance of their centroid and bounding box coordinates calculated by the two platforms. The EO area for mapped annotations calculated by the two platforms was not a perfect match [coefficient of determination (*R*^2^ = 0.98693)]; therefore, 325 annotations with an EO bin difference of >1 were filtered out (*R*^2^ = 0.99964). QuPath EO areas were used to calculate EO bins for morphological analyses. Measurements made within QuPath included Feret diameters, solidity, and circularity (*17*).

A proportion of the tissue sections analyzed in this study were designated as coming from a known pancreas region: head (PH), body (PB), tail (PT), or an unknown location (PO). The number of pancreas sections from each region varied because of the availability of archived tissue (tables S1 and S2). All EO metrics assessed were first calculated per WSI. Where necessary, the mean of multiple sections of the same pancreas region was calculated to generate pancreas region data. The mean of all pancreas regions measured was used to generate donor-level data for visualization. Data were visualized in R (4.1) using ggplot2, ggspatial, and ggh4x packages.

### Statistical analysis

Statistical analysis of data was performed in R (4.1) using the rstatix (0.7.2), emmeans (1.11.0), and car (v3.1.2) packages. Because the data presented are from a population study where *n* numbers cannot be controlled experimentally, statistical tests that allow for an unbalanced design were performed. The nonparametric Mann-Whitney *U* test was performed between two groups with a nonnormal distribution, followed by the “Benjamini-Hochberg” procedure to calculate adjusted *P* values.

Where three or more groups were compared across the same variable, the Kruskal-Wallis test was performed. Post hoc comparisons of all groups were performed using Tukey’s multiple-comparison test with the emmeans package (v1.11.0) in R.

The type “II” sum of squares analysis of variance (ANOVA) was performed with the car package (v3.1.2), where two or more groups are compared across multiple bin sizes or EO classes. Subsequent post hoc testing with Tukey’s multiple-comparison test was performed when comparing all selected groups to each other, whereas Dunnett’s test was used for comparing select groups to the control group of donors without diabetes. Specific contrasts computed are indicated in the legend, and all statistical test results can be found in data S1. Significance values are denoted as *P < 0.05, **P < 0.01, and ***P < 0.001.

## Supplementary Materials

### The PDF file includes:

EXE-T1D consortium members Figs. S1 to S13 Legends for tables S1 to S5 Legend for data S1
