# Supplementary material for: Small things matter: Lack of extraislet β cells in type 1 diabetes
Source: Sci Adv. 2025 Nov 12;11(46):eadz2251. doi: 10.1126/sciadv.adz2251 (PMC12609120; doi:10.1126/sciadv.adz2251)
Supplement: Supplementary file 1 — EXE-T1D consortium members Figs. S1 to S13 Legends for tables S1 to S5 Legend for data S1 [file sciadv.adz2251_sm.pdf]

Science Advances
AAAS

# Supplementary Materials for

## Small things matter: Lack of extraislet β cells in type 1 diabetes

Kathryn Murrall *et al.*

Corresponding author: Sarah J. Richardson, s.richardson@exeter.ac.uk

*Sci. Adv.* **11**, eadz2251 (2025)
DOI: 10.1126/sciadv.adz2251

**The PDF file includes:**

EXE-T1D consortium members
Figs. S1 to S13
Legends for tables S1 to S5
Legend for data S1

**Other Supplementary Material for this manuscript includes the following:**

Tables S1 to S5
Data S1

**EXE-T1D consortium members:**

Amber M. Luckett[1], Rebecca A Dobbs[1], Clara Domingo-Vila[2], Kathleen M Gillespie[3], Andrew T Hattersley[1], Michelle Hudson[1], Timothy J McDonald[1], Noel G Morgan[1], Kathryn Murrall[1], Sarah J Richardson[1], Megan E Smithmyer[4], Cate Speake[4], Timothy IM Tree[2], Bart Roep[5], William A Hagopian[6], Ben Blaise[7,8], Iain Yardley[8], Matthew B Johnson[1], Richard Oram[1.]

1. Department of Clinical & Biomedical Sciences, University of Exeter Medical School, Exeter, UK

2. Department of Immunobiology, School of Immunology & Microbial Sciences (SIMS), King's College, London, UK

3. Translational Health Sciences, Bristol Medical School, University of Bristol, Southmead Hospital, Bristol, UK

4. Center for Interventional Immunology, Benaroya Research Institute, Seattle, WA, USA

5. Department of Internal Medicine, Leiden University Medical Center, Leiden, the Netherlands

6. Indiana University School of Medicine, Department of Pediatrics, Indianapolis, USA

7. King's College London, Centre for the Developing Brain, St Thomas' Hospital, London, United Kingdom

8. Guy's and St Thomas' NHS Foundation Trust, Evelina London Children's Hospital, Department of Paediatric Surgery, London, United Kingdom

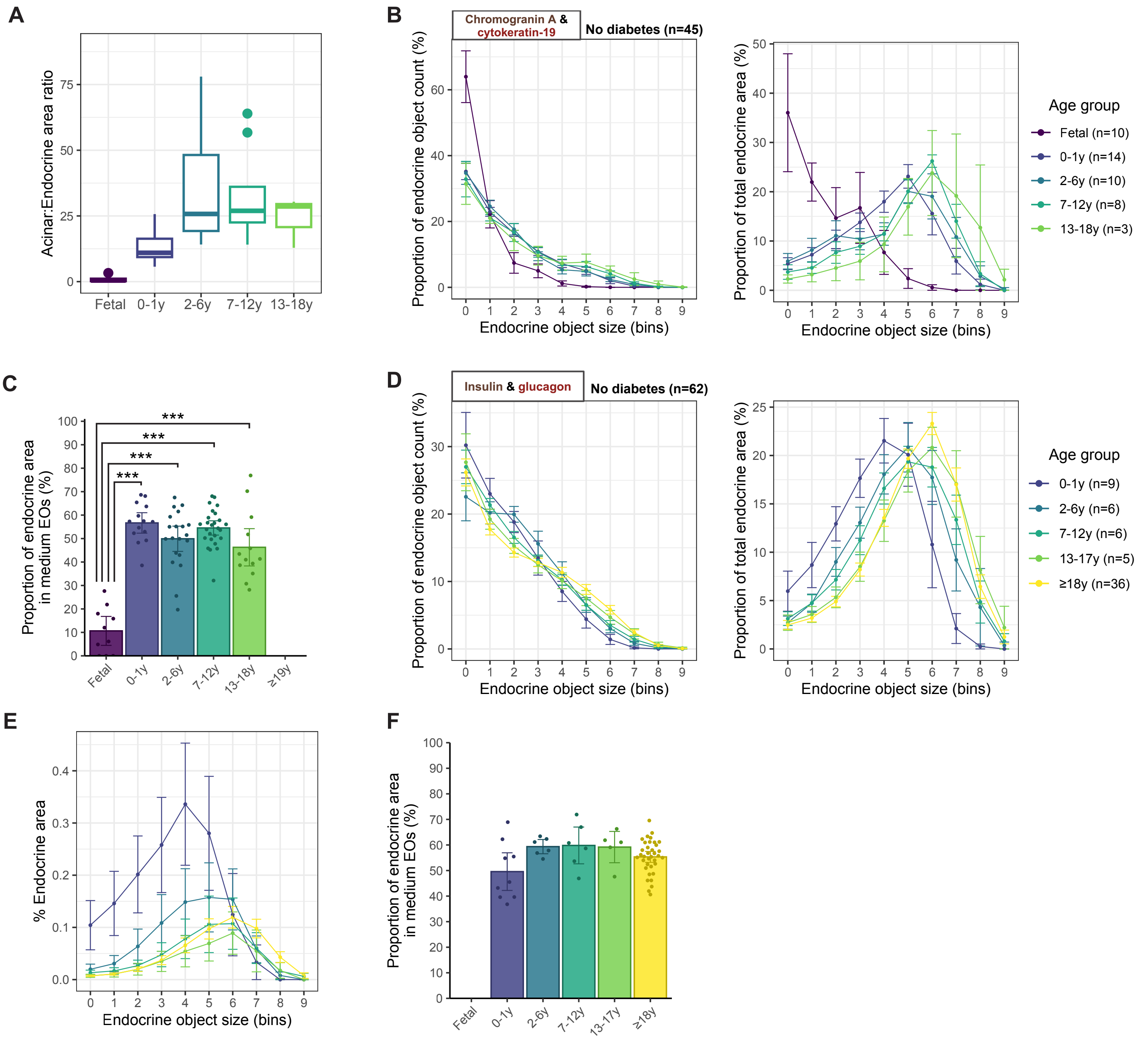


**Fig. S1. EO profile shifts towards larger objects with age in donors without diabetes, with the greatest changes occurring in the first few years post-birth. (A)** Box and whisker plot of Acinar:Endocrine area ratio in pancreata labelled for CgA/CK19 (n=45), grouped by age. **(B)** Proportion of EO count and endocrine area in each bin in pancreata labelled for CgA/CK19 and grouped by age. **(C)** Sum of the proportion of EO area in medium sized EOs (bins 4-6) for pancreata labelled for CgA/CK19. **(D)** Proportion of EO count and total endocrine area in each bin for pancreata labelled for Ins/Gluc (n=62), grouped by age. **(E)** % endocrine area in each EO bin in pancreata labelled for Ins/Gluc, grouped by age, from donors without diabetes. **(F)** Sum of the proportion of EO area in medium sized EOs (bins 4-6) in pancreata, grouped by age. Data are presented as mean or mean and scatter ± 95% CI. Kruskal-Wallis test followed by Tukey's *post hoc* test performed to calculate *p* values for **(C)** and **(F)**. *p <0.05, **p <0.01, ***p<0.001.

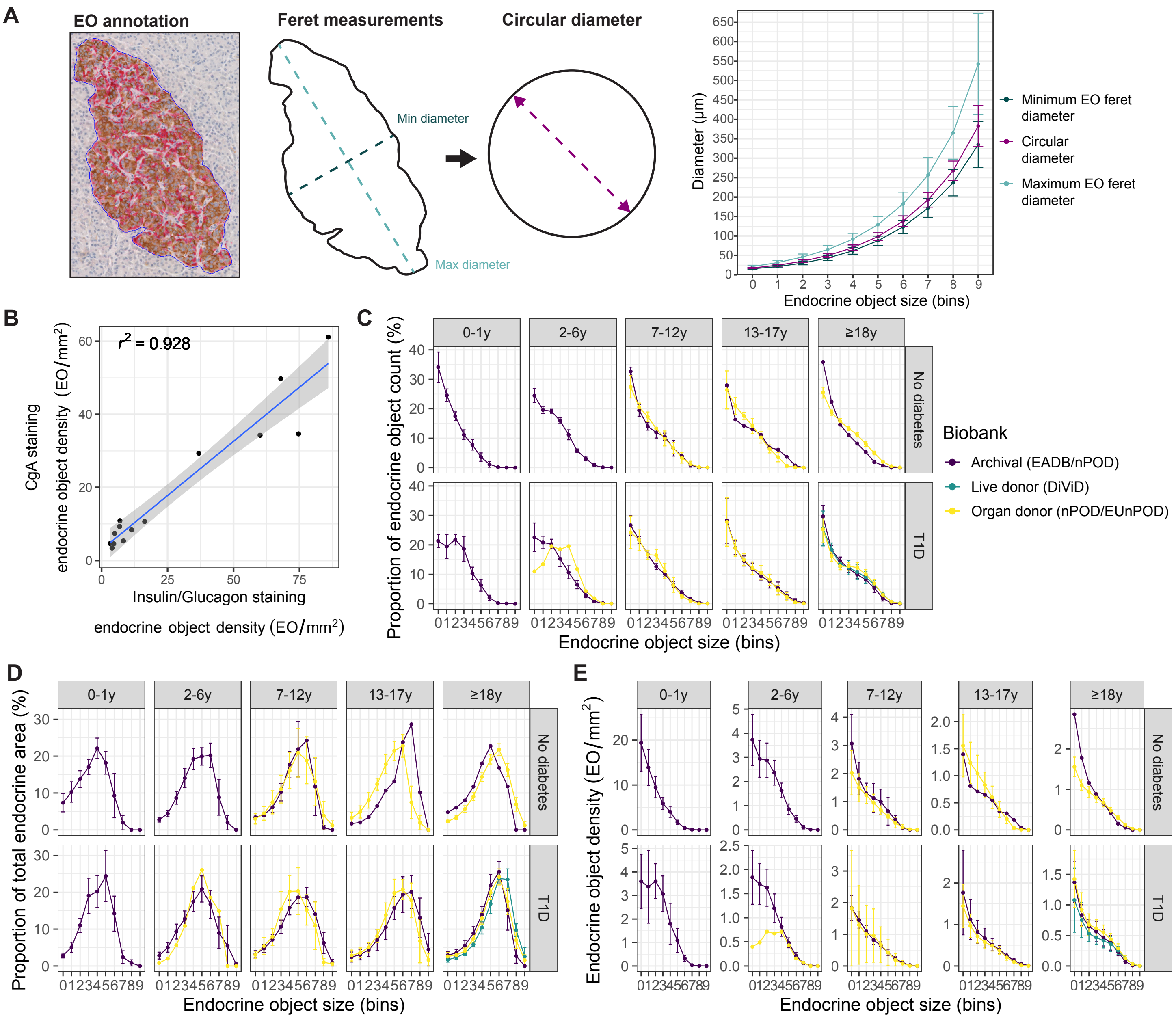


**Fig. S2**. **Quality control checks ensure confounding factors such as immunolabelling marker selection and tissue collection method do not significantly alter EO profiles. (A)** Schematic representing minimum and maximum Feret diameter measurements for an EO. Circular diameters were derived from the area of EOs. Data plotted show circular, minimum and maximum diameters for EOs in pancreata from donors without diabetes. Data are mean ± SD. **(B)** Scatter plot and linear regression comparing EO density in serially labelled chromogranin A/cytokeratin-19 (CgA/CK19) and insulin/glucagon (Ins/Gluc) sections. **(C-E)** Proportion of EO count **(C)**, proportion of endocrine area **(D)** and EO density **(E)** in bins in age matched donors with and without T1D, comparing whether collection method affects EO profile. Data are presented as mean ± 95% CI.

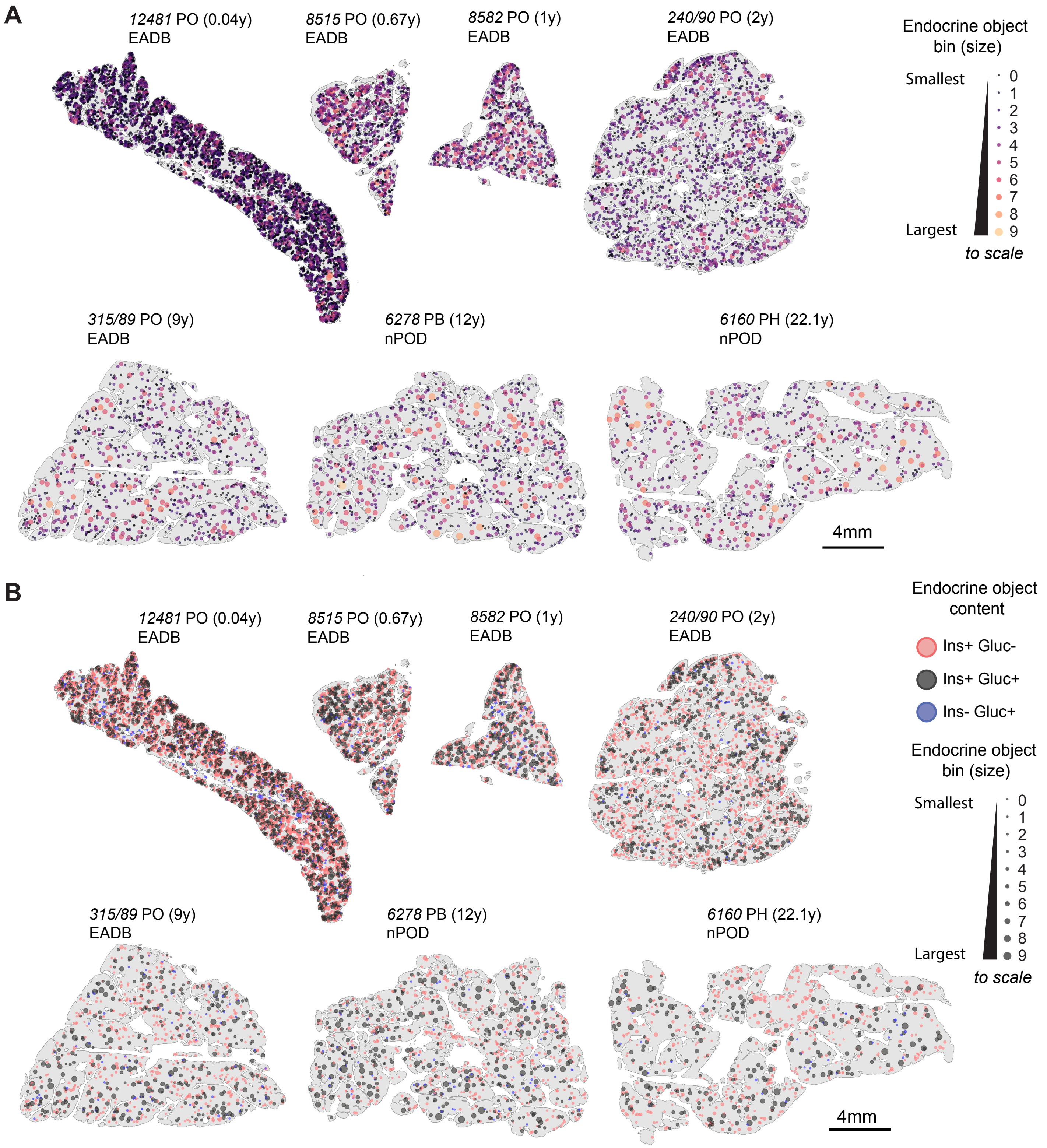


**Fig. S3. Representative spatial plots of the developing pancreas post-birth through to adulthood in donors without diabetes. (A and B)** Representative spatial plots showing development of endocrine architecture in the neonatal pancreas through to adulthood. Each EO is represented by a single point; size indicates the corresponding EO bin. The colours of the points designate either size, where the darker colours are smaller EOs and the lighter colours are larger EOs **(A)**, or endocrine content **(B)**. Each section is labelled with donor ID, pancreas location (PH: pancreas head; PB: pancreas body; PT: pancreas tail; PO: other/unknown), age (years) and biobank.

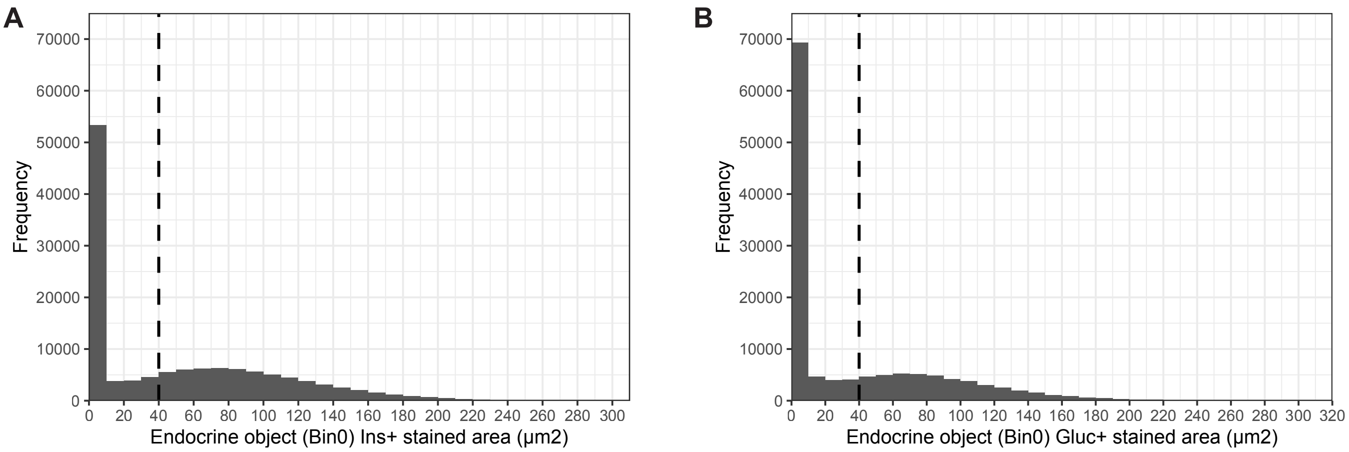


**Fig. S4. Endocrine objects demarcated by an AI-based classifier were selected for based on a positively stained insulin and glucagon area of >40 µm. Annotations below this threshold had little or low chromogen staining (A** and **B).** Insulin positive area **(A)** or glucagon positive area **(B)** per $\mu m^2$ in annotated objects within bin 0. *x* axis intercept is at 40 µm. Data show the frequency of annotations, with corresponding binned positively stained area.

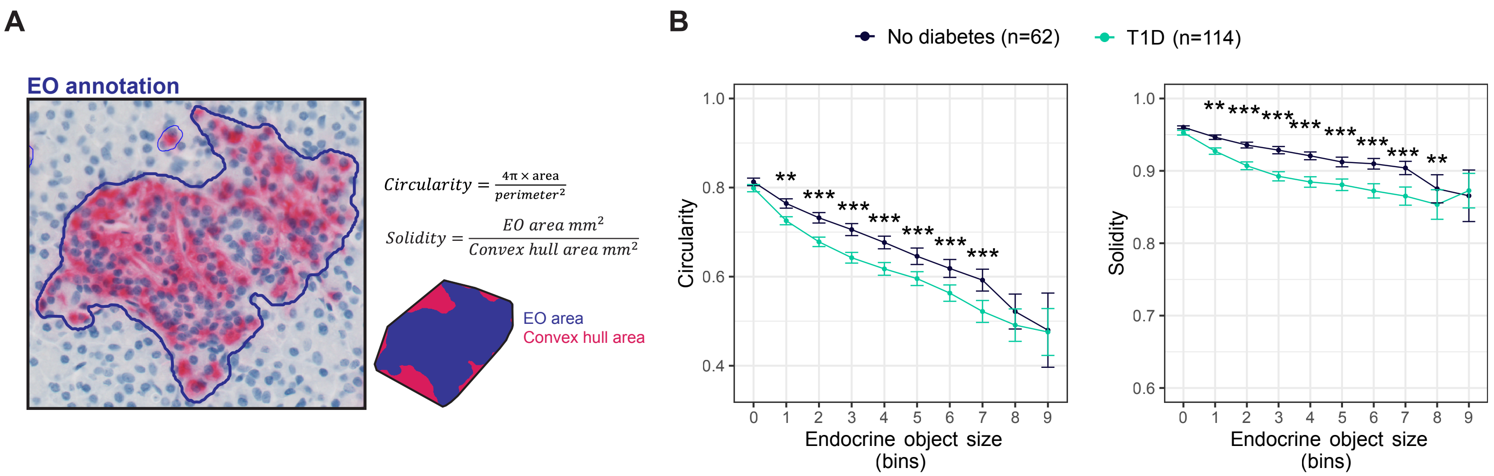


**Fig. S5. β-cells within larger endocrine objects in older individuals are more likely to persist in type 1 diabetes. (A)** Schematic showing the calculation of circularity and solidity for an EO. **(B)** Changes in circularity and solidity in EO bins for pancreata from donors with and without T1D. Type II ANOVA with Tukey's *post hoc* test was used to calculate *p* values when comparing No Diabetes to T1D for each bin. Data are presented as mean ± 95% CI. *p <0.05, **p <0.01, ***p<0.001.

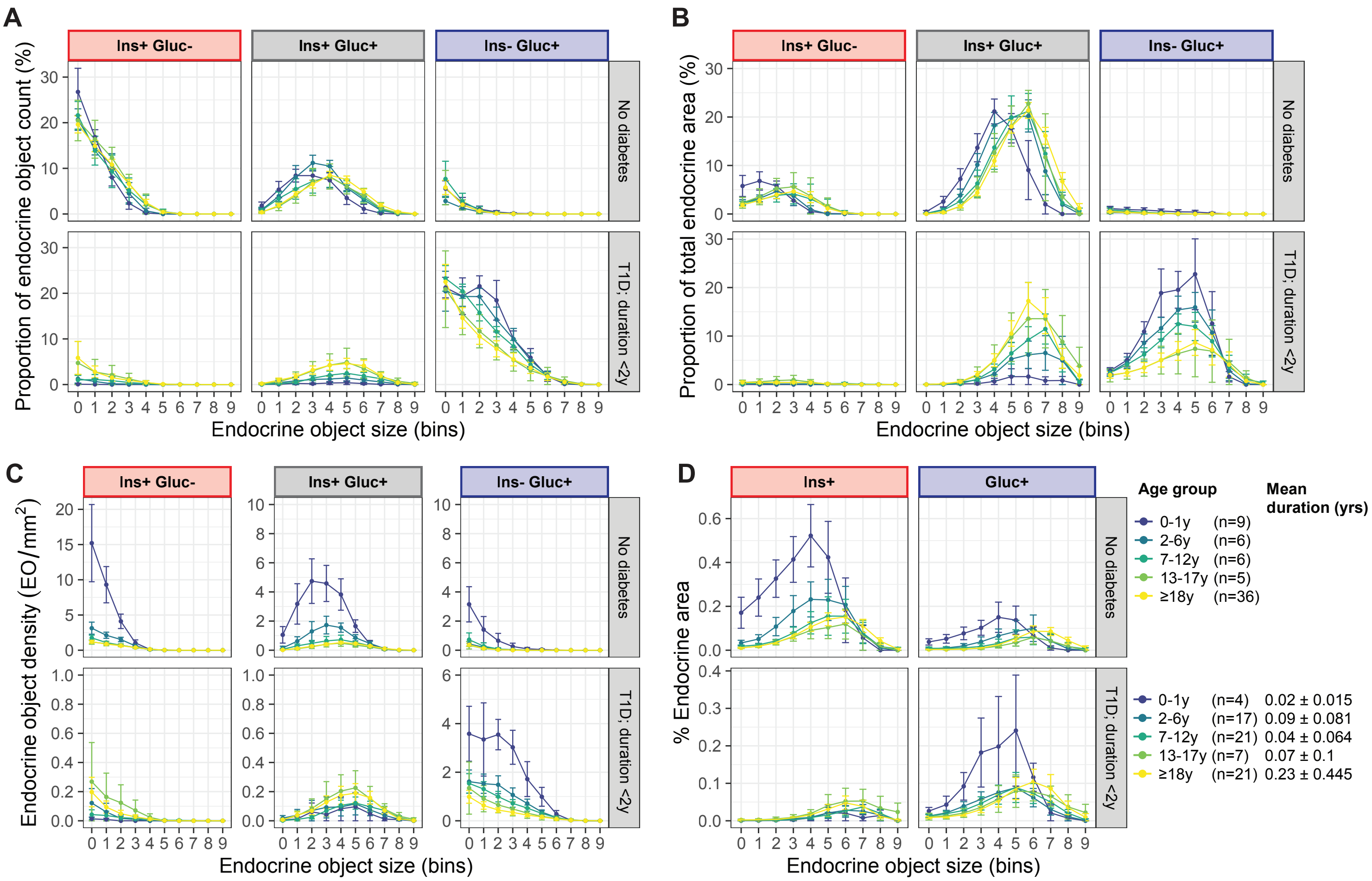


**Fig. S6. The contribution of EOs with different hormone content to total endocrine area, density, and hormone area as a proportion of tissue area are impacted by developmental stage in donors with and without T1D. (A and B)** Proportion of total EO count **(A)** and area **(B)** in each bin classified by endocrine content for donors with and without diabetes (T1D duration <2y), grouped by age. These data are also shown in Fig. 4A and B. **(C and D)** EO density **(C)** and % hormone-positive area as a proportion of tissue area **(D)** in bins classified by endocrine content for donors with and without T1D (T1D duration <2y), grouped by age. The mean diabetes duration ± SD for each of the T1D age groups is denoted. Data are presented as mean ±95% CI.

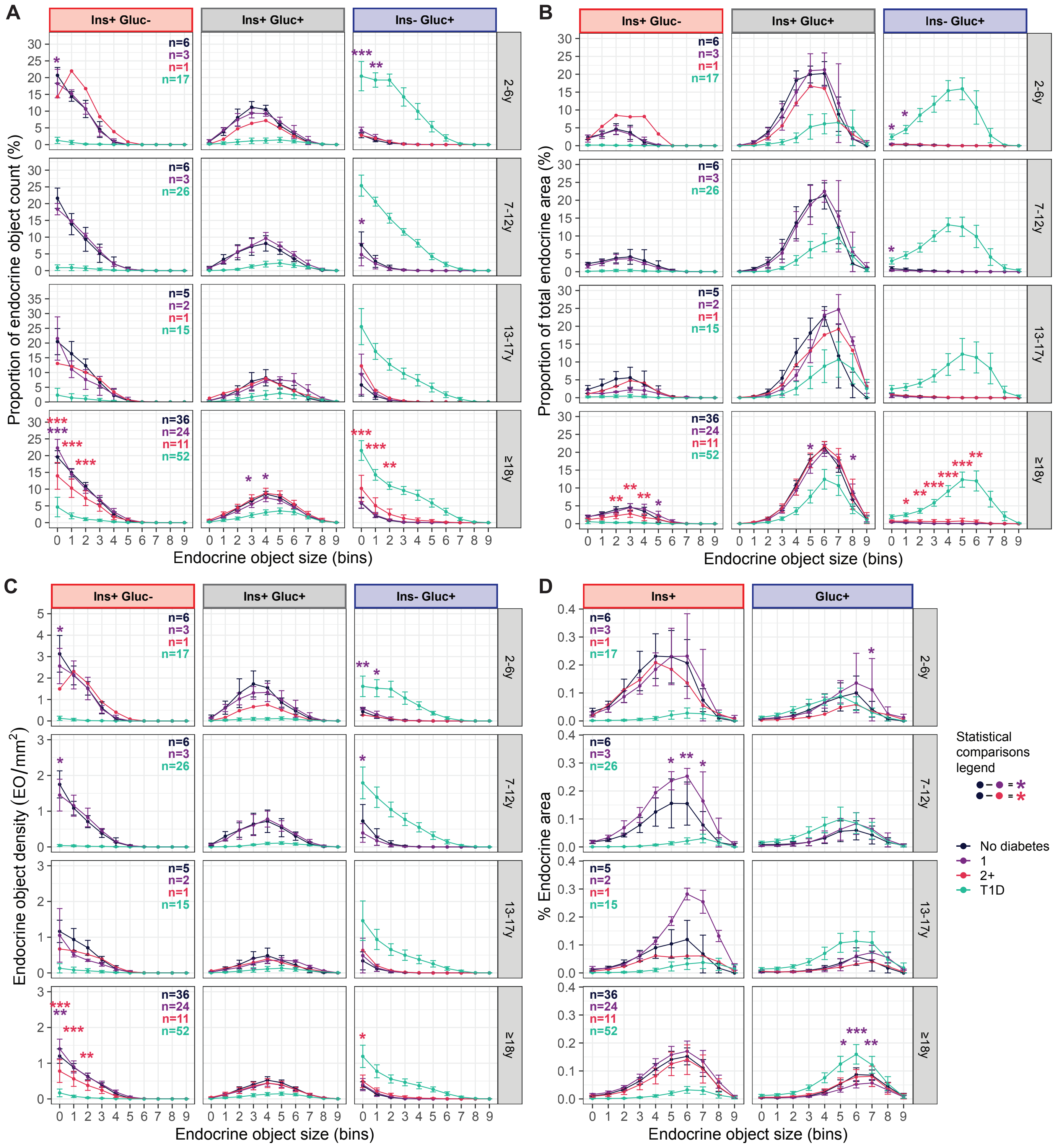

**Fig. S7. A subset of single and multiple autoantibody-positive donors display a reduction in small Ins+Gluc- EOs and elevation of Ins-Gluc+ EOs, however, the total endocrine area closely resembles that observed in pancreata from donors without diabetes. (A-C)** Proportion of total endocrine count **(A),** endocrine area **(B),** and EO density **(C)** classified by endocrine content and grouped by age for donors without diabetes, single and multiple autoantibody-positive donors and T1D donors. **(D)** Insulin or glucagon-labelled area as a proportion of tissue area in each EO bin. Type II ANOVA followed by Tukey *post hoc* comparing No Diabetes and T1D for each bin was performed to calculate $p$ values for groups with n ≥ 3. Data are presented as mean ±95% CI. *p <0.05, **p <0.01, ***p<0.001**.**

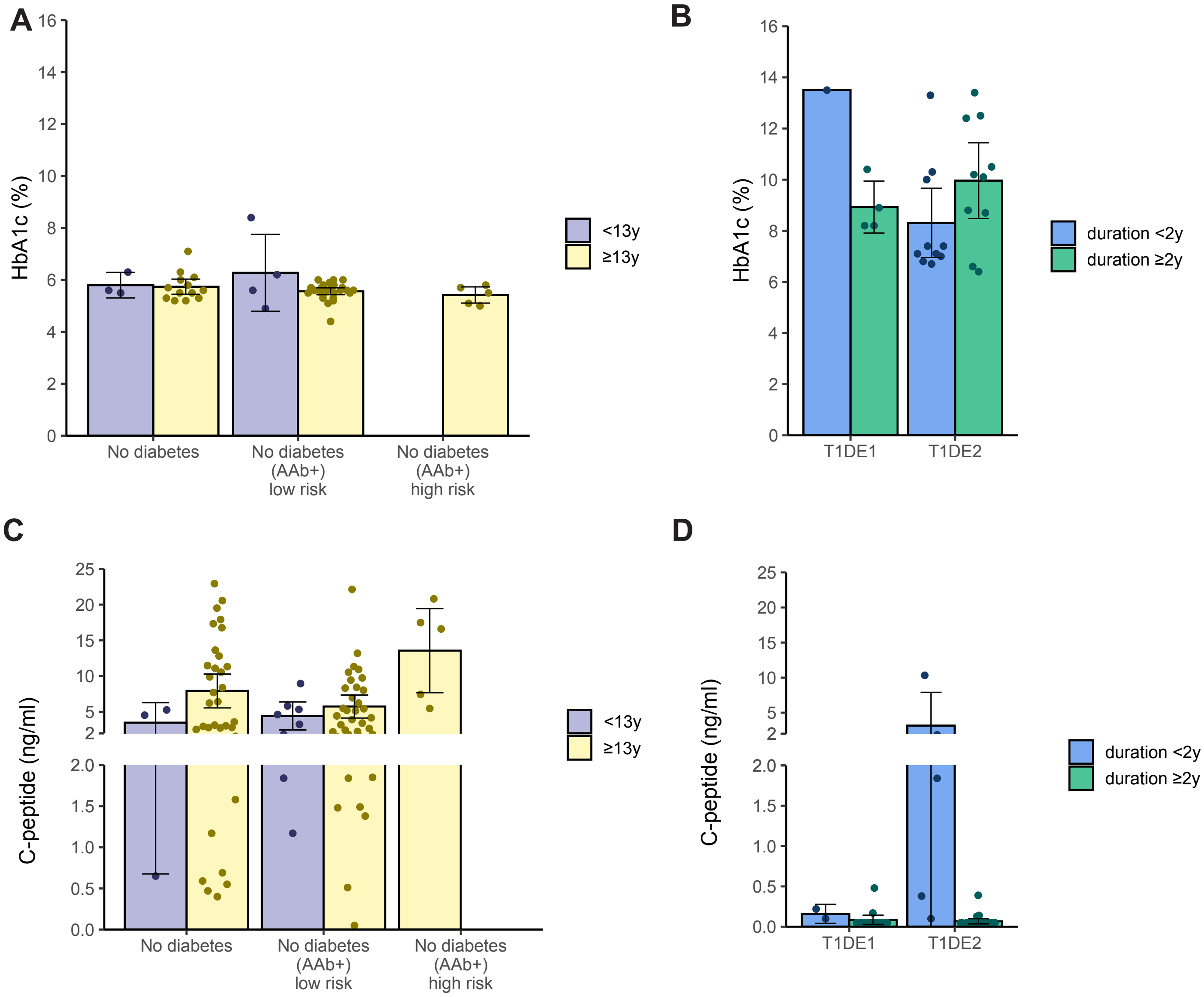


**Fig. S8. Available HbA1c and C-peptide levels for donors from the nPOD biobank which were also histologically assessed. (A)** HbA1c levels in low-risk and high-risk autoantibody-positive donors and donors without diabetes, grouped by age. High-risk autoantibody-positive donors were designated by meeting three or more of the following criteria: GRS1 or GRS2 above the 50$^{th}$ percentile, reported HLA hyperexpression, insulitis reported in the nPOD DataPortal and presence of IA2A+ autoantibody. **(B)** HbA1c levels for donors with T1D (T1DE1: diagnosed <13y; T1DE2: diagnosed ≥13y), with a disease duration of <2 or ≥2y. **(C)** C-peptide levels (ng/mL) for low and high-risk autoantibody-positive donors and donors without diabetes or autoantibodies, grouped by age. **(D)** C-peptide levels (ng/mL) for T1DE1 and T1DE2 donors separated by duration of disease. Summary data can be found in **tables S4** and **S5**. Data are mean and scatter ± 95% CI**.**

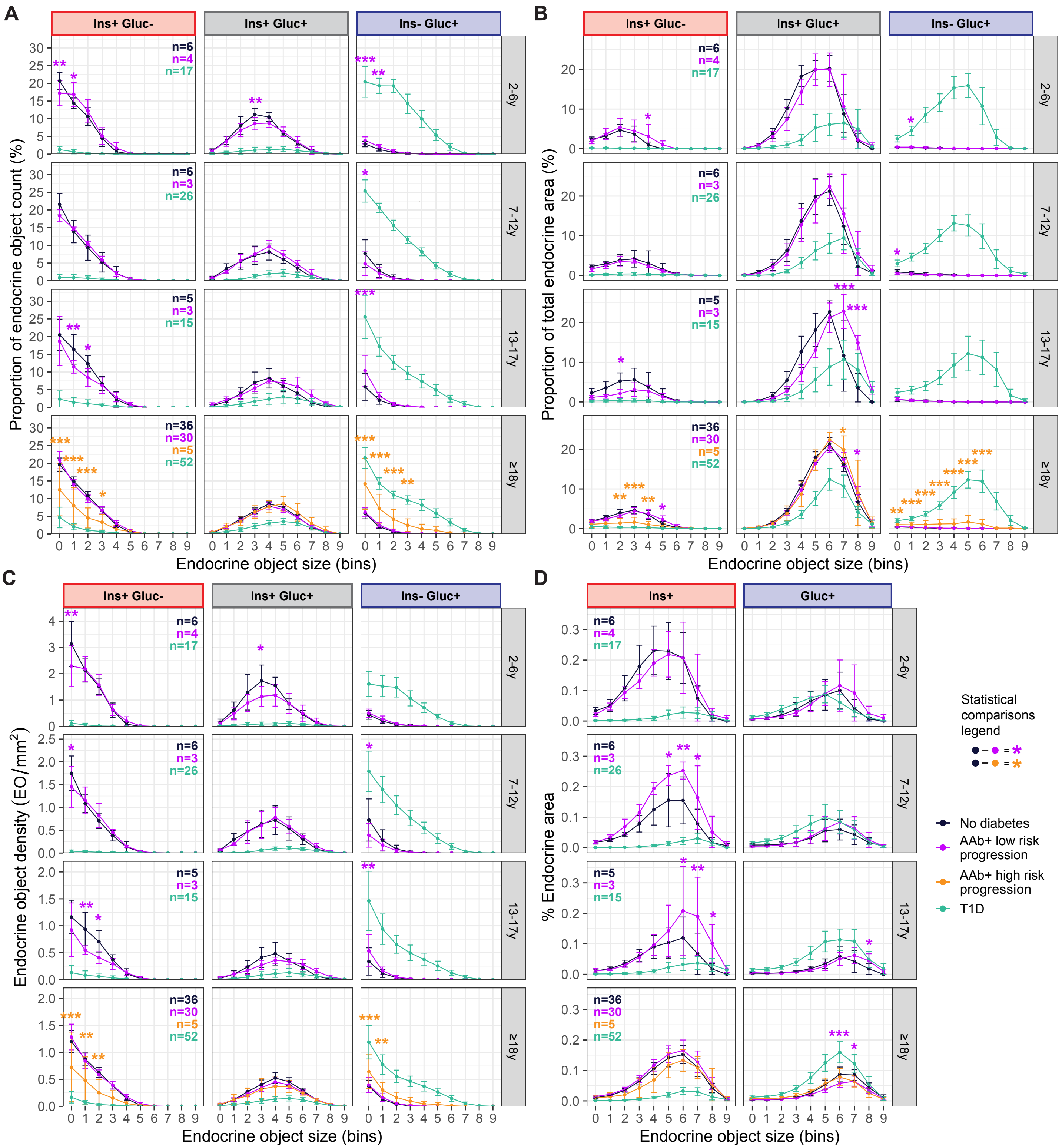

**Fig. S9. Pancreata from high-risk autoantibody-positive donors have markedly fewer Ins+Gluc- EOs and more Ins-Gluc+ EOs than donors without diabetes, but share a similar Ins+Gluc+ EO profile. (A-C)** Proportion of EO count **(A)**, endocrine area **(B)** and EO density **(C)** for each bin, separated by endocrine content, comparing pancreata from age-matched donors without diabetes, low-risk and high-risk autoantibody-positive donors, and donors with T1D. **(D)** % Insulin or glucagon area in EO bins, comparing age-matched low-risk and high-risk autoantibody-positive donors, donors without diabetes, and donors with T1D. Type II ANOVA followed

by Dunnett's *post hoc* comparing No Diabetes to low-risk, and No Diabetes to high risk, AAb+ donors for each bin was performed to calculate $p$ values for groups with $n \geq 3$. Data are mean and scatter ± 95% CI**.** Data used to designate AAb+ donor risk can be found in **table S1.**

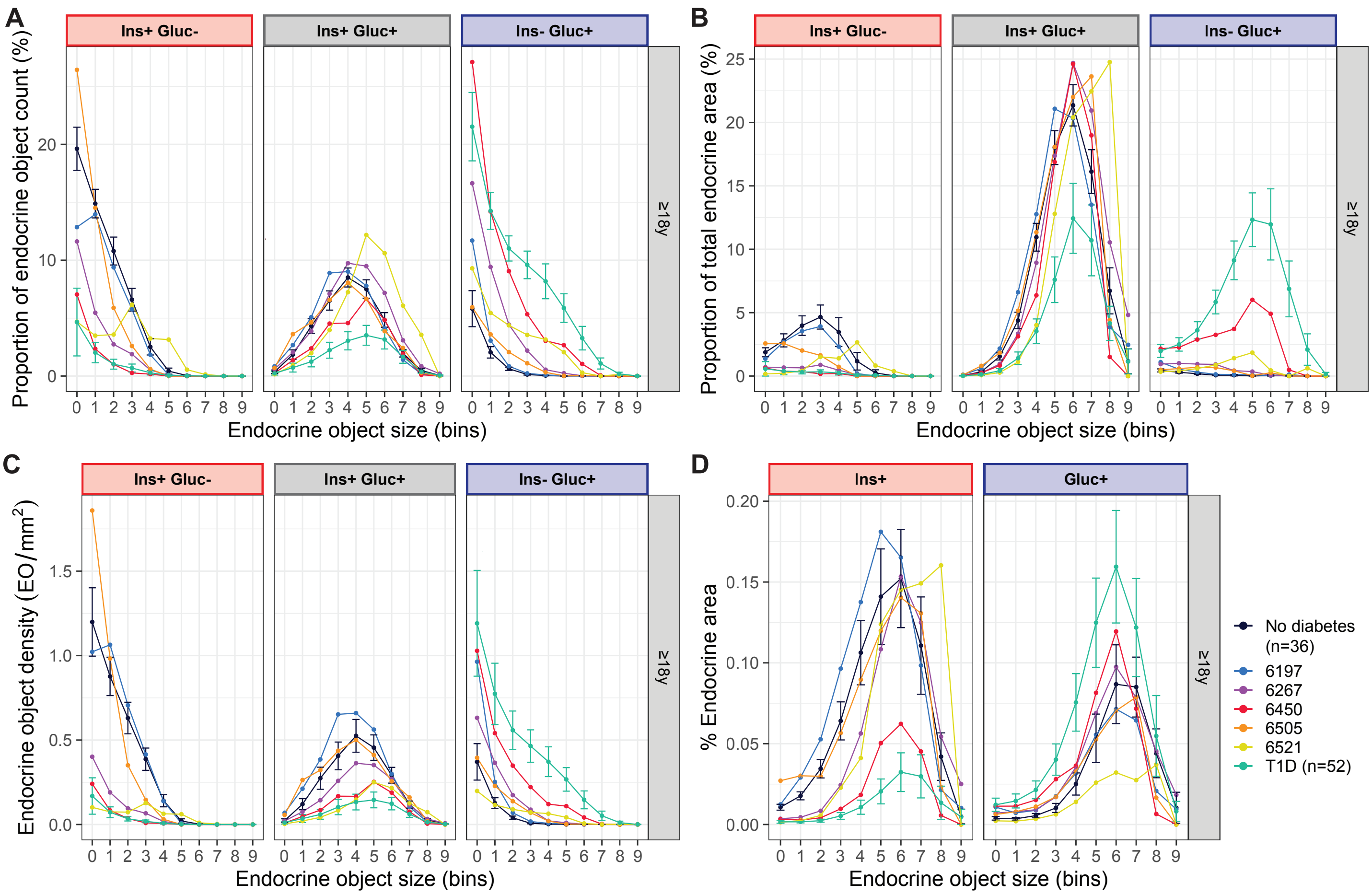


**Fig. S10. A subset of high-risk autoantibody-positive donors have markedly reduced small Ins+Gluc- EOs, determined by individual donor analysis. (A-C)** Proportion of EO count **(A)**, endocrine area **(B)** and EO density (also presented in Fig. 6B) **(C)** for each bin, separated by endocrine content, comparing pancreata from age-matched high-risk autoantibody-positive donors and donors without diabetes, and donors with T1D. **(D)** % Insulin or glucagon area in EO bins, comparing pancreata from age-matched high-risk autoantibody-positive donors and donors without diabetes, and donors with T1D. Data are presented as mean ± 95% CI for donors with and without T1D, or the mean of each bin for each high-risk autoantibody-positive donor.

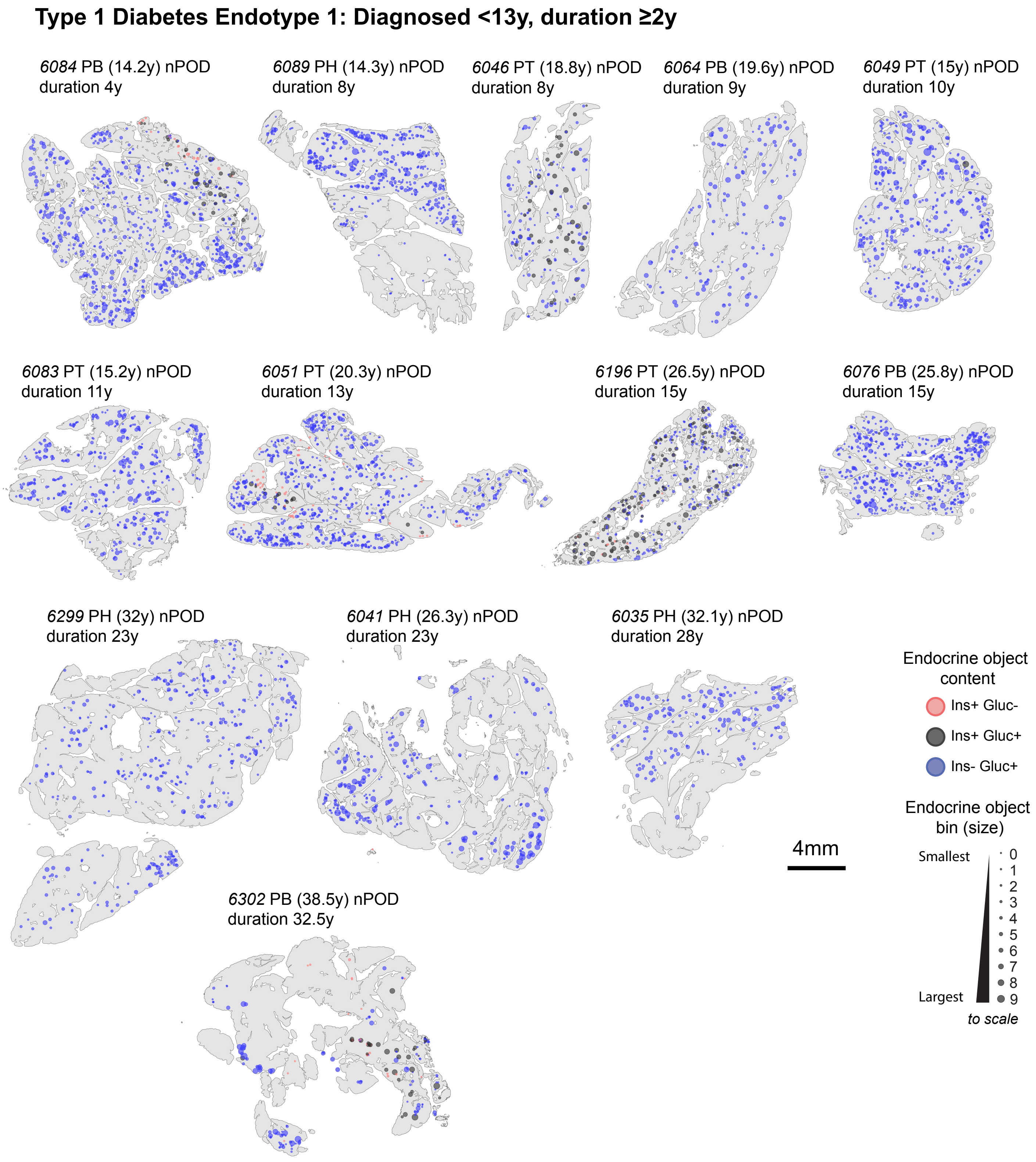


**Fig. S11. Spatial plots of all donors with type 1 diabetes aged ≥13y with a diabetes duration of ≥2y and a clinical onset of <13y)** Spatial plots of T1D donors aged ≥13y that were diagnosed <13y (T1DE1) with a diabetes duration of ≥2y, ordered by diabetes duration. Each EO is represented by a single point; size corresponds to EO bin, and colour denotes endocrine content. Each section is labelled with donor ID, pancreas location (PH: pancreas head; PB: pancreas body; PT: pancreas tail; PO: other/unknown), age (years), biobank and diabetes duration (years).

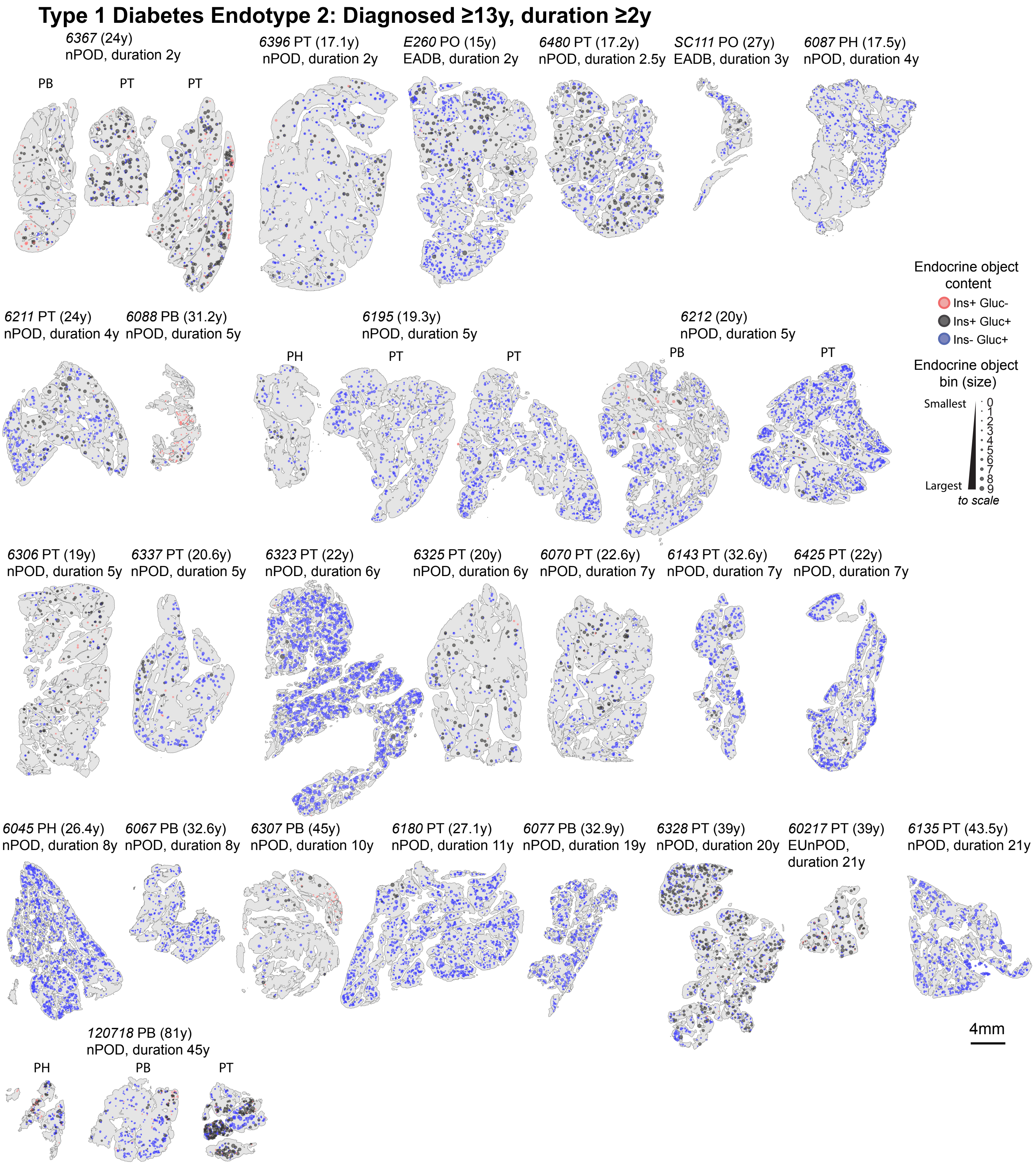


**Fig. S12. Spatial plots of all donors with type 1 diabetes aged ≥13y with a diabetes duration of ≥2y and a clinical onset of ≥13y show preservation of β-cells in larger glucagon-containing EOs in a subset of donors.** Spatial plots of donors with T1D aged ≥13y that were diagnosed ≥13y (T1DE2) with a diabetes duration of ≥2y, ordered by diabetes duration. Each EO is represented by a single point; size corresponds to EO bin and colour

denotes endocrine content. Each section is labelled with donor ID, pancreas location (PH: pancreas head; PB: pancreas body; PT, pancreas tail; PO: other/unknown), age (years), biobank and diabetes duration (years).

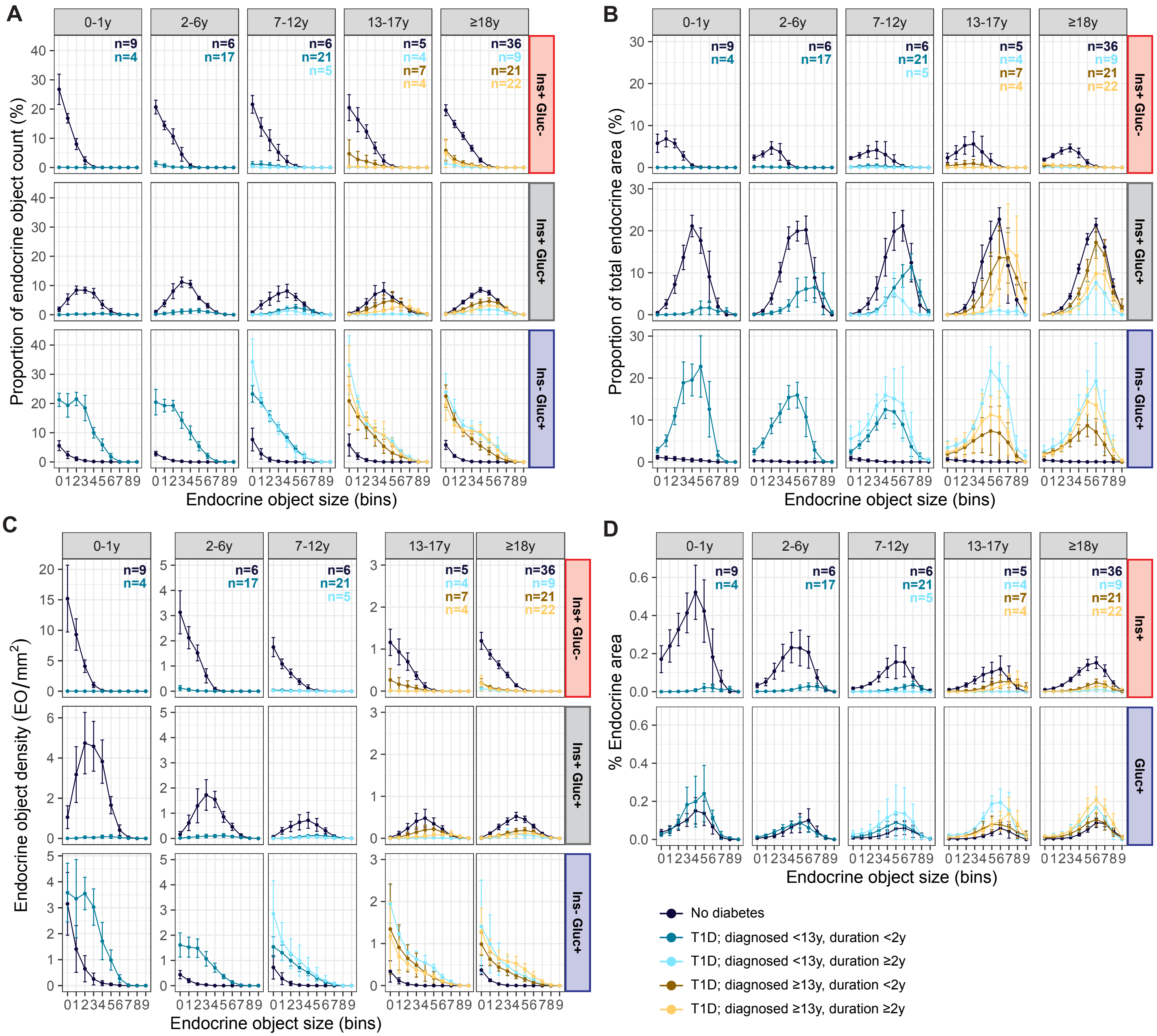


**Fig. S13. Characterisation of EO profile, density and % endocrine area in age- and duration-matched individuals, diagnosed <13y (T1DE1) or ≥13y (T1DE2), shows a profound absence of small Ins+ EOs across all ages in type 1 diabetes. (A-C)** Proportion of EO count **(A)**, endocrine area **(B)**, and EO density **(C)** for each bin separated by endocrine content, in age-matched donors without diabetes and donors with T1D, grouped by T1D endotype (T1DE1/2) and diabetes duration (≤2y or ≥2y). **(D)** % Insulin or glucagon area for each bin in pancreata from donors with and without T1D. Data are presented as mean ± 95% CI.

**Table S1. (separate file)**
Detailed donor information.

**Table S2. (separate file)**
List of pancreas sections for each donor included in the analyses presented in this paper, denoting whether the section was recently stained or obtained from an archive.

**Table S3. (separate file)**
Changes in the proportion of endocrine area within small (bin 0-3) and large (bin 7-9) EOs with age.

**Table S4. (separate file)**
HbA1c information obtained from the nPOD data portal for donors included in this study, where available.

**Table S5. (separate file)**
C-peptide levels obtained from the nPOD data portal for donors included in this study, where available.

**Data S1. (separate file)**
Statistical summary.
